# Cucumber (*Cucumis sativus* L.) Nitric Oxide Synthase Associated Gene1 (*CsNOA1*) Plays a Role in Chilling Stress

**DOI:** 10.3389/fpls.2016.01652

**Published:** 2016-11-11

**Authors:** Xingwang Liu, Bin Liu, Shudan Xue, Yanlinq Cai, Wenzhu Qi, Chen Jian, Shuo Xu, Ting Wang, Huazhong Ren

**Affiliations:** ^1^College of Horticulture and Developmental Regulation for Protected Vegetable Crops, China Agricultural UniversityBeijing, China; ^2^Beijing Key Laboratory of Growth and Developmental Regulation for Protected Vegetable Crops, China Agricultural UniversityBeijing, China

**Keywords:** cucumber, nitric oxide, *CsNOA1*, nitrate reductase, transgenic plants, chilling stress

## Abstract

Nitric oxide (NO) is a gaseous signaling molecule in plants, transducing information as a result of exposure to low temperatures. However, the underlying molecular mechanism linking NO with chilling stress is not well understood. Here, we functionally characterized the cucumber (*Cucumis sativus L*.) nitric oxide synthase-associated gene, *NITRIC OXIDE ASSOCIATED 1* (*CsNOA1*). Expression analysis of *CsNOA1*, using quantitative real-time PCR, *in situ* hybridization, and a promoter::β-glucuronidase (GUS) reporter assay, revealed that it is expressed mainly in the root and shoot apical meristem (SAM), and that expression is up-regulated by low temperatures. A CsNOA1-GFP fusion protein was found to be localized in the mitochondria, and ectopic expression of *CsNOA1* in the *A. thaliana noa1* mutant partially rescued the normal phenotype. When overexpressing *CsNOA1* in the *Atnoa1* mutant under normal condition, no obvious phenotypic differences was observed between its wild type and transgenic plants. However, the leaves from mutant plant grown under chilling conditions showed hydrophanous spots and wilting. Physiology tolerance markers, chlorophyll fluorescence parameter (Fv/Fm), and electrolyte leakage, were observed to dramatically change, compared mutant to overexpressing lines. Transgenic cucumber plants revealed that the gene is required by seedlings to tolerate chilling stress: constitutive over-expression of *CsNOA1* led to a greater accumulation of soluble sugars, starch, and an up-regulation of Cold-regulatory C-repeat binding factor3 (*CBF3*) expression as well as a lower chilling damage index (CI). Conversely, suppression of *CsNOA1* expression resulted in the opposite phenotype and a reduced NO content compared to wild type plants. Those results suggest that *CsNOA1* regulates cucumber seedlings chilling tolerance. Additionally, under normal condition, we took several classic inhibitors to perform, and detect endogenous NO levels in wild type cucumber seedling. The results suggest that generation of endogenous NO in cucumber leaves occurs largely independently in the (CsNOA1) and nitrate reductase (NR) pathway.

## Introduction

Plants are frequently exposed to adverse environmental conditions that can limit growth and development, among which low temperature is a key factor. Chilling stress can results in poor seed germination, stunted seedling growth, delayed crop heading, and increased pollen sterility (Xiong and Zhu, 2001; Beck et al., 2004; Minami et al., 2005). To withstand chilling damage, plants have developed multiple survival strategies. For example, upon exposure to cold stress, a set of signals are immediately triggered, including the calcium and reactive oxygen species burst, which activates the MAPK signal cascade, and ultimately initiates the downstream cold-responsive transcriptional cascade (Cheng et al., 2015). In addition, plants can set up physiological and biochemical adaptations to cope with the stress challenge (Ruelland et al., 2009; Theocharis et al., 2012). These adaptations include changes in membrane composition, the induction of anti-oxidative systems, and the synthesis of protective molecules. In addition, it has been reported that transcriptional regulation of the carbohydrate metabolic pathway *Arabidopsis thaliana* is essential for the accumulation of specific carbohydrates that represent an important factor in improved tolerance to chilling stress (Cook et al., 2004; Maruyama et al., 2009, 2014). Previous studies have also provided evidence for cold exposure triggering a large remodeling of plant metabolism, which, at least partly, depended on modifications in gene expression (Ruelland et al., 2009; Theocharis et al., 2012). Consequently there is interest in identifying and characterizing the signaling network underlying the low temperature stress and there is growing evidence that nitric oxide (NO) is an important signal for transducing information related to low temperature exposure.

A number of studies have shown that NO can alleviate low-temperature stress (Zhao et al., 2009; Liu et al., 2011; Yang et al., 2011a; Tan et al., 2013a). Additionally, Zhao et al. (2009) reported that cold acclimation in *A. thaliana* was associated with an increase in endogenous NO production, and Guillas et al. (2011) observed an immediate increase in NO synthesis in response to cold stress, which regulated the expression of cold-responsive genes, as well as with novel downstream elements identified as phosphosphingolipid metabolic species. In mammals, NO is synthesized via an oxidative mechanism involving NO synthase (NOS) enzymes, which oxidize arginine to generate citrulline and NO (Mayer and Hemmens, 1997; Wendehenne et al., 2001). In the algal species, *Ostrococcus tauri*, a NOS-like enzyme has been reported to synthesize NO (Foresi et al., 2010). Although NOS activity has been detected in plants, a novel plant NOS has not yet been identified. The *Arabidopsis thaliana* NO synthase1 (*AtNOS1*) was initially documented as a putative plant NOS, but was later renamed *AtNOA1* (*Arabidopsis thaliana NO-ASSOCIATED PROTEIN 1*), as it was suggested as a circular permuted GTPase of YIqF family (Moreau et al., 2008). The defective arginine-dependent NO synthesis activity in recombinant *AtNOS1* protein and the contradictory NO accumulation responses in NOA1-silenced mutants have confirmed that AtNOS1 is not an authentic NOS (Kwan et al., 2014). The indirect regulatory effect of NOA1 in NO production was demonstrated with impaired NO accumulation in NOA1-silenced *A. thaliana* (Guo et al., 2003). While others have suggested that the enzyme, nitrate reductase (NR), is more important to product NO in plants (Rockel et al., 2002; Planchet et al., 2005; Stöhr and Stremlau, 2006; Wilson et al., 2008). It has been reported that the *A. thaliana* double NR mutant, *nia1nia2*, fails to accumulate NO, or to mediate certain NO responses (Desikan et al., 2002). Moreover, NR inhibitors, such as sodium azide and tungstate have been shown to inhibit NO production in plants (Bright et al., 2006; Sang et al., 2008). Less well explored is the possibility that reductive pathways for NO production include the mitochondrial electron transport system and the enzyme, xanthine dehydrogenase/oxidase (Gupta and Kaiser, 2010). In addition, under aerobic conditions, exogenous hydroxylamine application to NR-deficient tobacco cell cultures resulted in the release of NO (Rümer et al., 2009).

We previously used exogenous application of an NO donor to elucidate the role of NO under chilling stress in cucumber (Liu et al., 2011), a warm-season species that requires protection from cold temperatures (Kuk and Shin, 2007). However, the role of NO in chilling stress tolerance and the source(s) of endogenous NO in cucumber seedlings have still not been resolved and these questions are addressed in this current study.

## Materials and methods

### Plant material, growth conditions, and treatments

Cucumber (*Cucumis sativus* L.) line ZN407 (collected from Ren lab in China Agricultural University) seedlings were grown in a growth chamber under a 16/8 h and 25/18°C day/night photoperiod. *A. thaliana* wild type (WT) Col-0 and *Atnoa1* mutant (Columbia ecotype background) plants were kindly provided by the Zhang laboratory (Zhao et al., 2009). WT, *Atnoa1* and transgenic *CsNOA1* seedlings were grown at 22°C under a long-day (16 h of light/8 h of dark) photoperiod at 100 μmol m^−2^ s^−1^ with 50–70% relative humidity in turf substrate. For chilling stress, 7-week-old seedlings were subjected to a 10 day chilling regime at 4°C.

Cucumber seedlings at the three leaf stage were used for the experimental treatments (Liu et al., 2011). Sodium nitroprusside (SNP, Sigma, USA) was used as an NO donor and the potassium salt of 2-(4-carboxyphenyl)-4, 4, 5, 5-tetramethylimidazoline-1-oxyl-3-oxide (cPTIO, Sigma, USA) was used as an NO scavenger. Sodium azide (NaN_3_) and tungstate were used as NR inhibitors and N^G^-Nitro-L-arginine Methyl Ester (L-NAME, Selleck, USA) and Nω-Nitro-L-arginine (L-NNA, Selleck, USA) as NOA1 inhibitors. Specific doses of NO donor, scavenger and inhibitors were sprayed onto the leaves of cucumber seedlings before exposure to 72 h of cold treatment at 4°C under continuous light (520 μmolm^−2^ s^−1^). Each treatment was repeated at least three times using 10 seedlings per treatment. After treatment, the third leaves were collected and stored at −80°C until further use.

### Endogenous distribution of cellular NO

The cellular distribution of endogenous NO in cucumber leaf sections (1.5 × 2.0 cm) was detected using confocal laser scanning microscopy (Nikon A1R-si) in combination with the fluorescent dye, 4-amino-5-methylamino-2,′7′-difluorofluoresceindiacetate (DAF-FM DA, Calbiochem) according to Zhao et al. (2009) with slight modifications. Briefly, after washing the excised leaves with buffer solution (20 mM HEPES-NaOH, pH 7.4), they were incubated in a buffer solution containing 25 μM DAF-FM DA for 1 h at 25°C. The incubated leaves were visualized using laser confocal scanning microscope after having been washed thoroughly with buffer solution to remove excess fluorophore. Excitation was at 488 nm and emission was at 515 nm.

### Quantification of endogenous NO

The concentration of endogenous NO was determined according to Zhao et al. (2009) with modifications. Fresh cucumber leaves (500 mg) were incubated with 100 U of Catalase (EC1.11.1.6, CAT) and 100 U of Superoxide Dismutase (EC1.15.1.1, SOD) for 5 min to remove endogenous reactive oxygen species (ROS) before addition of 5 ml oxyhaemoglobin (5 mM). After a 3 min incubation, the absorbance of the solution at 577 nm and 591 nm were used to calculate the amounts of oxyhaemoglobin and methaemoglobin, respectively, and the endogenous NO content was calculated based on the conversion ratio between oxyhaemoglobin and methaemoglobin.

### NR activity assay

NR activity was assayed based on a modified protocol from Rockel et al. (2002). One gram of cucumber leaf tissue was ground in liquid N_2_ and re-suspended in an extraction buffer containing 100 mM HEPES-KOH pH 7.5, 1 mM EDTA, 15% glycerol (v/v), 10 mM dithiothreitol, 0.1% Triton X-100, 1 mM phenylmethylsulfonyl fluoride, 20 μM FAD, 1 μM leupeptin, 5 μM Na_2_MoO_4_. The samples were centrifuged at 12,000 *g* for 15 min at 4°C. Five-hundred μl of each sample was then added to 250 μl of 1% (w/v) sulfanilamide (3 N HCl) and 250 μl 0.02% (w/v) N-(1-naphthyl) ethylene diamine and samples left for 20 min at room temperature. The samples were centrifuged at 16,000 *g* for 10 min, and the nitrite formed was assessed by colorimetric determination at OD546 nm using the Tecan Infinite M1000 plate reader. Absorbance was normalized to the protein concentration of the sample, as determined by the bicinchoninic acid (BCA) protein assay (ThermoFisher Scientific), using bovine serum albumin as a standard. To test whether NR could produce NO *in vitro*, previously published methods were used (Rockel et al., 2002).

### Determination of CsNOA1 activity

NOA1 activity determination was performed according to Zhao et al. (2007b). Approximately 1 g of leaf tissue, together with 50 mg of polyvinylpolypyrrolidone, were ground in liquid N_2_ and then re-suspended in extraction buffer (50 mM Tris-HCl, pH 7.4, 1 mM EDTA, 320 mM Sucrose, 1 mM dithiothreitol, 1 μM leupeptin, 1 μM pepstatin, and 1 mM phenylmethylsulfonyl fluoride). After centrifugation at 10,000 *g* for 30 min at 4°C, the supernatant was used for NOA1 activity determination using the citrulline assay and the NOA1 assay kit (Cayman Chemical). The reaction mixture (50 μl) contained 25 mM Tris-HCl, pH7.4, 3 μM tetrahydrobiopterin, 1 μM FAD, 1 μM FMN, 1 mM NADPH, 0.6 mM CaCl_2_, 0.1 μM Calmodulin, 0.3 μM (1 μl Ci) [^3^H] Arg (Amersham Biosciences), and 10 mL enzyme extract. After incubation for 30 min at 37°C, the reaction was stopped with 400 μl stop buffer (50 mM HEPES, pH 5.5, 5 mM EDTA). A 100 μl resin slur was added to the reaction mixture and the resin was removed by centrifugation at 10,000 *g*. Flow through (400 μl) was added to 5 mL of scintillation liquid and radioactivity was counted (LS 6000, Beckman). The protein contents in the supernatant were determined according to the Bradford method (1976) with bovine serum albumin as a standard.

### Transcriptional analysis by real time RT-PCR and semi-quantitative PCR

Total RNA was extracted from cucumber third true leaves using the Promega SV Total RNA Isolation System (http://promega.bioon.com.cn/), and cDNA was synthesized using MultiScribe™ reverse transcriptase (Applied Biosystems). Quantitative real-time RT-PCR was performed using SYBR®*Premix Ex Taq*™ from TaKaRa (China) and an Applied Bio-systems 7500 real-time PCR system. To determine the relative fold differences in template abundance for each sample, the −2^−ΔΔCt^ method was used. For semi-quantitative PCR, the conditions were as follows: 10 min at 94°C, then 25 cycles of 30 s at 94°C, 30 s at 50°C and 30s at 72°C, followed by 7 min at 72°C. The RT-PCR products were separated using a 2.0% (m/v) agarose gel. The gene specific primers are presented in Supplementary Table 3.

### *In situ* hybridization assay

The shoot apices of 10-day-old seedlings and roots from greenhouse grown plants were fixed, embedded, sectioned, and hybridized as described by Zhang et al. (2013). Digoxigenin-labeled sense and antisense RNA probes were generated using SP6 and T7 RNA polymerases (Roche), respectively, and PCR amplification. The primer pairs are listed in Supplementary Table 3.

### Transformation of *A. thaliana* and cucumber

To generate the *CsNOA1* over-expressing cucumber plants, the full length *CsNOA1* cDNA was cloned and inserted into the pCAMBIA1305.1 vector between *Xba*I (5′end) and *Sma*I (3′end) restriction sites (Zhang et al., 2014). To generate the *CsNOA1*-RNAi transgenic lines, two *CsNOA1* fragments were amplified using specific primers containing *Asc*I (5′end) and *Swa*I (3′end) endonuclease sites, and *Spe*I (5′end) and *BamH*I (3end) endonuclease sites. Two fragments were inversely inserted into the pFGC1008 vector (Zhang et al., 2014). For subcellular localization of CsNOA1, the coding region of *CsNOA1* without the stop codon was cloned and fused upstream of the EGFP (enhanced green fluorescent protein) sequence between the *Sai*I and *Kpn*I sites of the pEZS-NL vector to generate 35S:CsNOA1:GFP. To make the *CsNOA1*:GUS (β-glucuronidase) construct, a 1.8 kb sequence upstream from the ATG start site of the *CsNOA1* coding sequence was cloned and inserted into the PBI121 vector between the *Xba*I and *Bam H*I sites. *A. thaliana* transformation was performed as previously described (Clough and Bent, 1998), using the *Agrobacterium tumefaciens* strain GV3101. For cucumber transformation, all the resulting constructs and corresponding empty vectors were introduced into *A*. *tumefaciens* strain LBA4404 by electroporation and cucumber line NZ407 was transformed using the cotyledon transformation method (Zhang et al., 2014). Primers used are listed in Supplementary Table 3.

### Subcellular localization

Stable expression of the GFP control, CsNOA1-GFP, and Mito-tracker Red fusion proteins in T1 transgenic plants was examined and imaged using a LSM510 META confocal microscope (Zeiss). Measurements of the GFP and blue fluorescence of the chloroplasts were acquired with a 488-nm laser excitation and a band pass emission filter of 490–530 nm (GFP channel) and 650–750 nm (blue channel). For co-localization experiments with Mito-tracker Red and CsNOA1-GFP, multi-tracks were configured with the GFP channel setting as above and an additional red channel with an excitation of 543 nm and a band pass emission filter of 560–650 nm (red channel). Images of the GFP control and CsNOA1-GFP/Mito-tracker Red were collected using the same settings.

### GUS histochemical assay

GUS staining of the WT/*Pro*_*CsNOA*1_*:GUS* transgenic line (T2) was performed as previously described with slight modifications (Jefferson et al., 1987). Briefly, fresh tissue samples were fixed in ice cold 90% (v/v) acetone for 1 h and then vacuum filtrated for 30 min. Samples were vacuum infiltrated with staining buffer (50 mM PO_4_ buffer with 0.2% [v/v] tritonX-100, 100 mM K_3_Fe(CN)_6_, and 100 mMK_4_Fe(CN)_6_ for10 min on ice followed by vacuum infiltration in staining buffer containing 2 mM 5-bromo-4chloro-3-indolyl-ß-D-glucuronic acid for 15 min on ice. Samples were then incubated at 37°C for 30 min to 24 h followed by a wash with 75% (v/v) ethanol. The stained tissues were viewed under a stereo microscope and photographed.

### Transmission electron microscopy

Young leaves from transgenic plants and wild type were fixed in 2.5% (w/v) glutaraldehyde and rinsed thoroughly with a 0.1 M phosphate buffer (pH 6.8). Samples were post-fixed with 1% osmic acid, washed in 0.1 M phosphate buffer (pH 6.8), dehydrated through an acetone series (using the sequence 30, 50, 70, 80, 90, and 100%), and then embedded in Spurr's resin. Thin cross-sections were made with a UC6I microtome (Leica) and examined with a JEM-123O scanning transmission electron microscope (Liu et al., 2016).

### Electrolyte leakage assay

Electrolyte leakage was assayed according to Liu et al. (2011). Briefly, tubes containing six to eight *A. thaliana* leaves, detached from 20-days old plants and chilled at 4°C for 10 days, and cucumber leaves detached from 25-days old plants and chilled at 4°C for 72 h, were placed in a low-temperature bath (Grant) set at 0°C. The bath temperature was lowered at a rate of 2°C h^−1^. Tubes were then remove at the defined temperatures and thawed overnight at 4°C in the dark, before incubation with 5 mL of deionized water at 25°C for 2 h with gentle shaking (150 rpm). Electrical conductivity in the bathing solution was first determined (C1), before samples were heated to 100°C for 30 min and the second electrical conductivity (C2) of the bathing solution determined. Relative ion leakage was expressed as a percentage of the total conductivity after heating to 100°C. Relative ion leakage % = C1/C2 × 100.

### Determination of starch, soluble sugars, and proline and the iodine staining assay

Starch and total soluble sugar content in cucumber leaves were measured as previously described (Liu et al., 2011). Proline accumulation in cucumber leaves was determined as method previously described (Zhao et al., 2009) using L-Pro as standard. Briefly, leaves from the lines to be tested were harvested, weighed and extracted in 3% sulfosalicylic acid. An aliquot of each extract (2 mL) was incubated with 2 mL of ninhydrin reagent (2.5% [w/v] ninhydrin, 60% [v/v] glacial acetic acid, 40% 6 M phosphoric acid) and 2 mL of glacial acetic acid at 100°C for 45 min, and the reaction was terminated in an ice bath. Toluene (5 mL) was added, followed by vortexing and incubation at 23°C for 24 h. The absorbance was measured at 520 nm. For the iodine staining assay, cucumber leaves were harvested at the end of the light phase (24 h without darkness) from plants grown for 5 weeks in a chamber as described above. Different doses of NO donor, scavenger, and inhibitors were sprayed onto the leaves of cucumber seedling before exposing them in to an I_2_/IK solution for 24 h and then destaining for 15 min in water (Zhang et al., 2008).

### Statistical analysis

All data were obtained from at least three independent experiments with three replicates each. Data were analyzed using one-way analysis of variance (ANOVA) followed by Duncan's multiple range test (*P* < 0.05).

### Fv/Fm assay and chilling damage index

The chilling damage index was measured following the methods described by Liu et al. (2011). The Fv/Fm was measured using a portable fluorometer (FMS2; Hansatech, Kings's Lynn, UK). Cucumber and *Arabidopsis thaliana* leaves were dark-acclimated for 30 min before Fv/Fm measurement. The maximal efficiency of PSII photochemistry in the dark-acclimated leaves was calculated according to the formula: Fv/Fm = (Fm-Fo)/Fm.

## Results

### Identification of the cucumber *CsNOA1* gene

**Figure 1:**
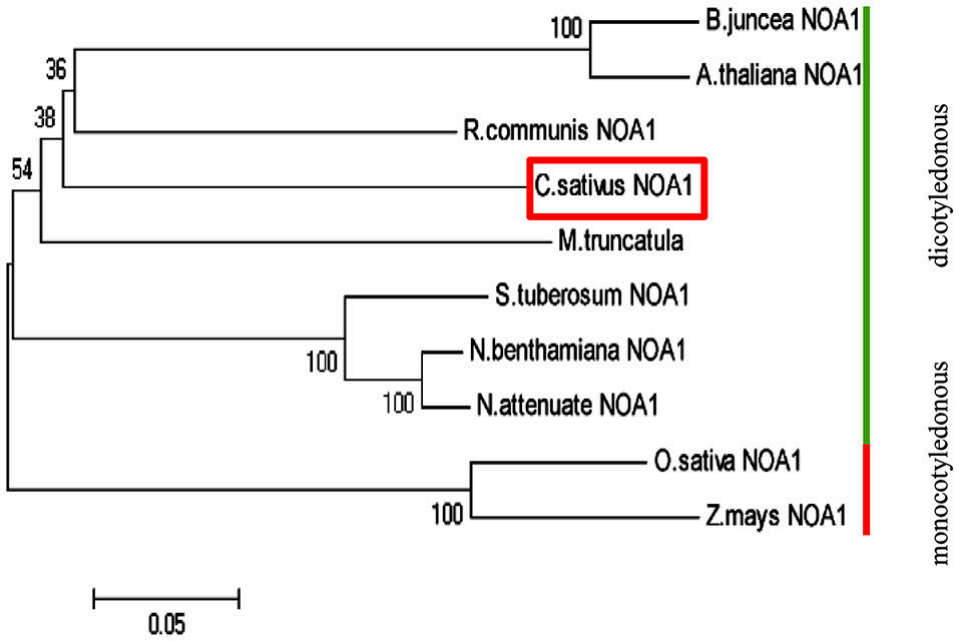
**Phylogenetic analysis of NOA1 homologs in various species**. This phylogenetic tree was constructed using the Neighbor—Joining (NJ) method through MEGA 5.0 software. Ten species were used for this analysis and formed two main groups: dicotyledonous group and monocotyledon group. A NOA1 homolog from cucumber is indicated in the red box. Gene ID for each of the NOA1 protein used for this analysis is listed in the “accession numbers.”

Basic Local Alignment Search Tool (BLAST) analysis of the Cucumber Genome Database (Huang et al., 2009) revealed a single *NOA1-like* gene, named *CsNOA1* (*Csa5M168870*). *CsNOA1* has 12 exons and 11 introns, which is different to the *A. thaliana* homolog, *AtNOA1*, which contains 13 exons, and 12 introns. The full-length *CsNOA1* cDNA is predicted to encode a protein of 556 amino acids (Supplementary Figure S1A). An alignment of the CsNOA1 amino acid sequence with apparent NOA1 homologs from other plant species (Supplementary Figure S1B) showed that CsNOA1, AtNOA1, and OsNOA1 (from rice, *Oryza sativa*) share three domains that are typical of the GTPase family: the zinc-binding domain (ZBD), the circularly permuted G-domain (CPG), and the C-terminal domain (CTD) (Moreau et al., 2008; Sudhamsu et al., 2008; Anand et al., 2009) (Supplementary Figure S1B). Over the full length sequence, CsNOA1 shows 66 and 64% identity to AtNOA1 and OsNOA1, respectively, indicating that the NOA1 sequence is well conserved between monocots and dicots. To better understand the evolutionary relationship between CsNOA1 and other NOA1 homologs, a phylogenetic analysis was performed using the neighbor-joining (NJ) method (Saitou and Nei, 1987; Figure 1). The phylogenetic tree divides the NOA1 homologs into two clades: the dicotyledon (green line) and monocotyledon (red line) group, with CsNOA1 in the dicotyledon clade as expected.

### Low temperature increases the expression of *CsNOA1*

**Figure 2:**
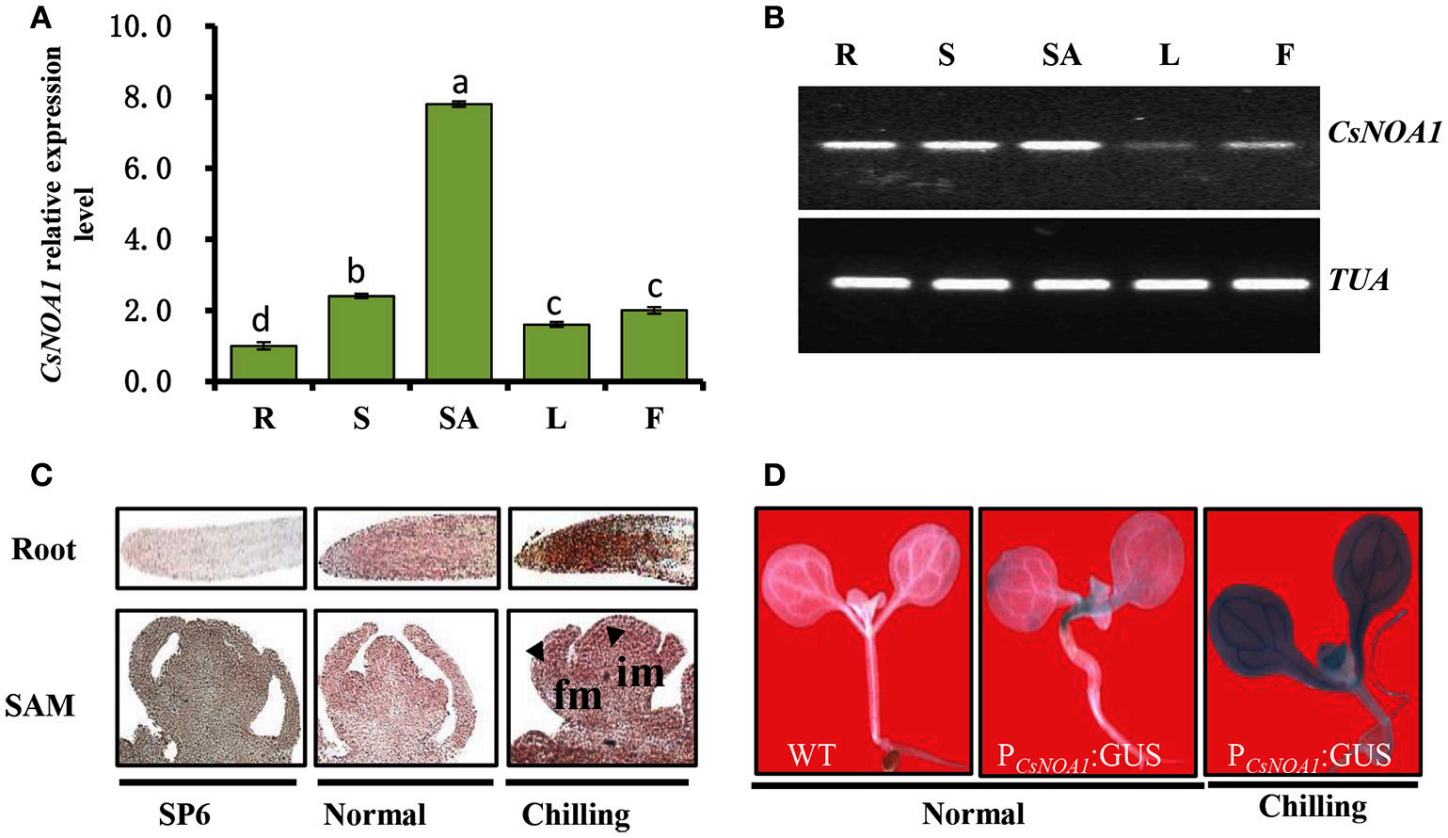
***CsNOA1* expression profile analyses. (A)** Relative expression of *CsNOA1* in cucumber tissues. R, root; S, stem; SA, shoot apical; L, leaf; F, flower. **(B)** Semi-quantitative RT-PCR analyses of *CsNOA1* expression in cucumber, the cucumberα*-TUBULIN* (*TUA*) was used as an internal control, and three biological replicates were performed for these experiments. **(C)** *In situ* hybridization of *CsNOA1* in plants grown under normal and chilling conditions. Hybridization with the *CsNOA1* sense probe gave no signal in the SP6 control. im, inflorescence meristem; fm, floral meristem. **(D)** Transgenic plants (10 days-old seedlings) harboring Pro_*CsNOA*1_:*GUS* were stained for GUS activity in seedlings under normal and chilling condition. The mean values of three independent samples and standard errors are presented; the same letter above the column indicates no significant difference at *P* < 0.05.

To obtain insights into the biological function of *CsNAO1*, its expression was examined in different organs with highest levels in the shoot apex (Figures 2A,B). Since exogenous NO has been shown to alleviate chilling stress injury in cucumber seedlings (Liu et al., 2011), we hypothesized that *CsNOA1* may be regulated by low temperatures. *CsNOA1* mRNA was detected by *in situ* hybridization analysis throughout the inflorescence meristem and floral meristem, as shown in Figure 2C, with higher expression in the im, fm and root apex under chilling conditions. We also analyzed *CsNOA1* expression using a *CsNOA1* promoter::GUS transcriptional fusion and histochemical staining. GUS labeling was strongest in the root and stem (Figure 2D) and the expression in these organs was further enhanced when plants were grown at low temperatures, indicating an induction of CsNOA1 expression under these conditions.

### Subcellular localization of CsNOA1

**Figure 3:**
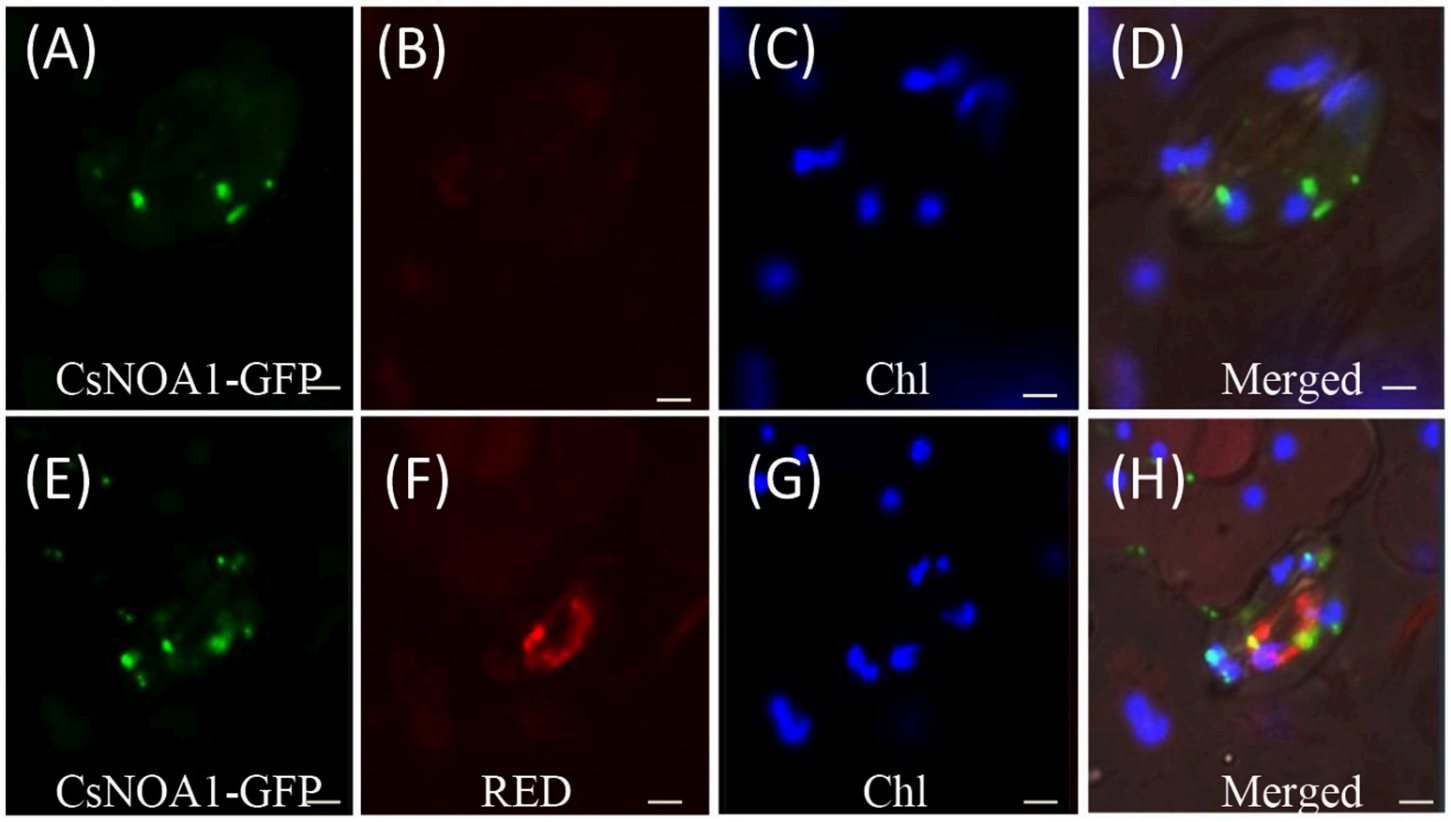
**Subcellular localization of CsNOA1 protein cucumber leaf stomata**. **(A)** CsNOA1-GFP fusion protein was stably expressed in cucumber leaf stomata and observed by confocal laser scanning microscopy (as a control). **(B)** No Mito-tracker Red shown in **(A)** could be imaged using Red channel setting of LSM510 META. **(C)** Leaf stomata fluorescence (Chl) could be imaged using Blue channel setting ofLSM510 META. **(D)** Merged image of **(A–C)**. **(E)** CsNOA1-GFP fusion protein was stably expressed in cucumber leaf stomata and observed by confocal laser scanning microscopy. **(F)** Mito-tracker Red shown in **(E)** could be imaged using Red channel setting of LSM510 META. **(G)** Leaf stomata fluorescence could be imaged in **(E)** using Blue channel setting ofLSM510 META. **(H)** Merged image of **(E–G)**. Scale bar = 50 μm.

To determine the subcellular localization of CsNOA1, green fluorescent protein (GFP) fused to the full-length *CsNOA1* sequence was stably expressed in cucumber seedlings under the control of the 35S promoter. As shown in Figures 3A–D, GFP signal was observed in small subcellular vesicles in the guard cells, but did not overlap with the blue fluorescence of the chloroplasts, indicating that CsNOA1 may be localized in the mitochondria or Golgi apparatus. We therefore used a mitochondria-specific stain, Mito-tracker Red (Matre et al., 2009) to evaluate the *CsNOA1-GFP* transgenic lines (Figures 3E–H), and this analysis showed co-localization with the CsNOA1-GFP fusion protein (Figures 3E–H), suggesting that CsNOA1 is localized in the mitochondria. This is congruent with the previously reported subcellular localization of AtNOA1 and TaNOA1 from *A. thaliana* and wheat, respectively (Guo and Crawford, 2005; Hao et al., 2010).

### Ectopic expression of *CsNOA1* in *A. thaliana* results in increased chilling stress tolerance

**Figure 4:**
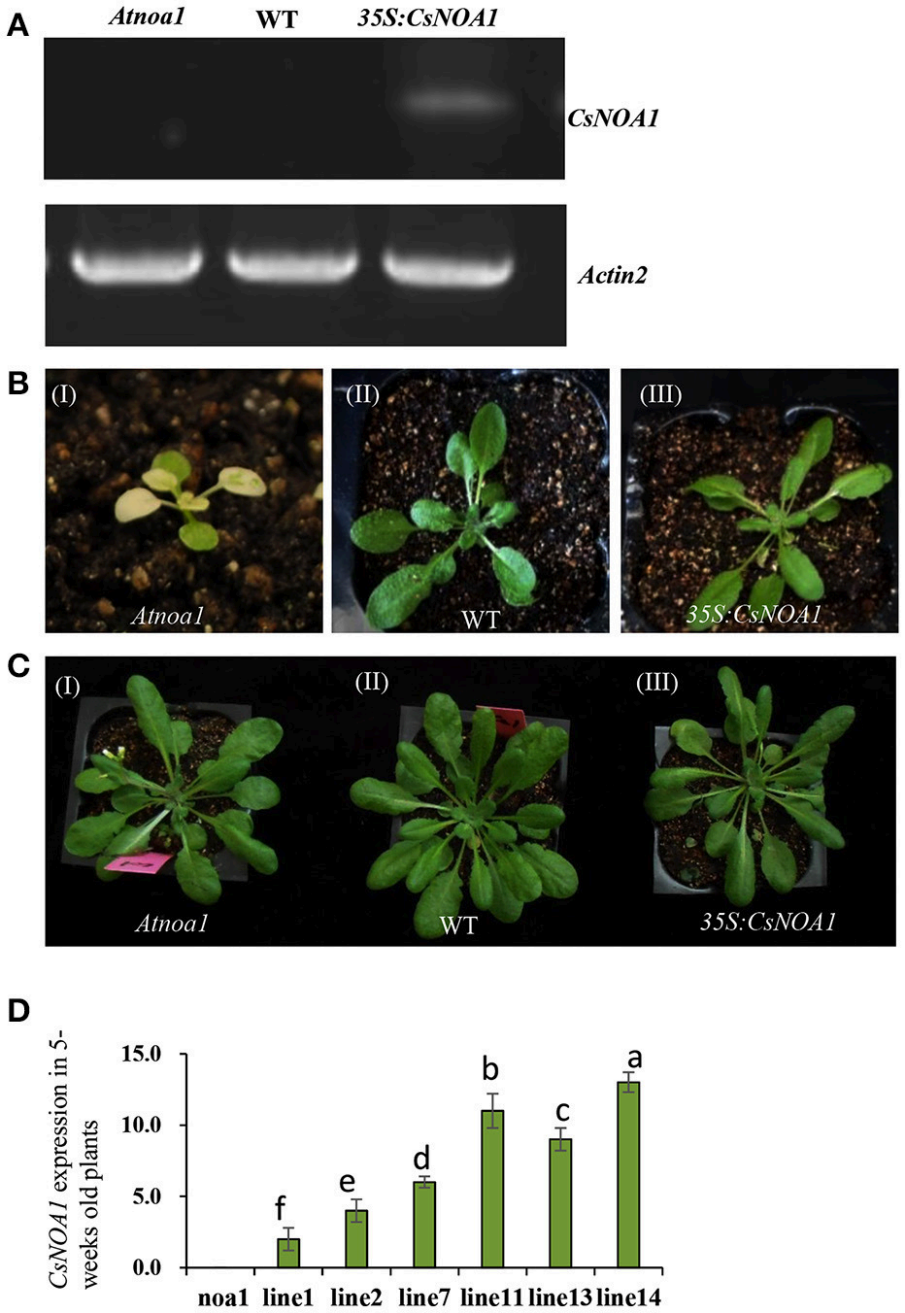
**Phenotypic analysis of *Arabidopsis thaliana* ectopically expressing *CsNOA1*. (A)** Semi-quantitative PCR analyses of *CsNOA1* in transgenic plants in the *Atnoa1* background. The *Arabidopsis thaliana ACTIN2* gene was used as an internal control. **(B)** Phenotype of transgenic *Arabidopsis thaliana* of the (I) *Atnoa1* mutant phenotype under normal conditions (20 days); (II) Wild type grown under the same conditions; (III) Ectopically expressing *CsNOA1* in the *Atnoa1* background. **(C)** Phenotype of transgenic *Arabidopsis thaliana* of the (I) *Atnoa1* mutant phenotype under normal conditions (7-weeks); (II)Wild type grown under the same conditions; (III) Ectopically expressing *CsNOA1* in the *Atnoa1* background. **(D)** Real time RT-PCR analyses of *CsNOA1* in six selected transgenic lines in the *noa1* background. The *Arabidopsis actin2* was used as an internal control, three biological replicates were performed for these experiments. Error bars indicate the standard errors. Different letters above the column indicate significant difference at *P* < 0.05.

To investigate the biological role of *CsNOA1*, we ectopically expressed the full-length *CsNOA1* cDNA under the control of a 35S promoter in an *Arabidopsis thaliana nitric oxide associated* 1 (*Atnoa1*) mutant. A total of 19 independent transgenic lines were obtained, all of which showed partial rescue of the phenotypes of the *Atnoa1* mutant similar phenotypes. As shown in Figure 4, normal plant size and green coloration of the leaves in transgenic plants were restored as compared with those in the *noa1* mutant (Figures 4A–C). We chose six transgenic lines to analyze expression of *CsNOA1* and determined that it was expressed at different levels in these lines (Figure 4D).

**Figure 5:**
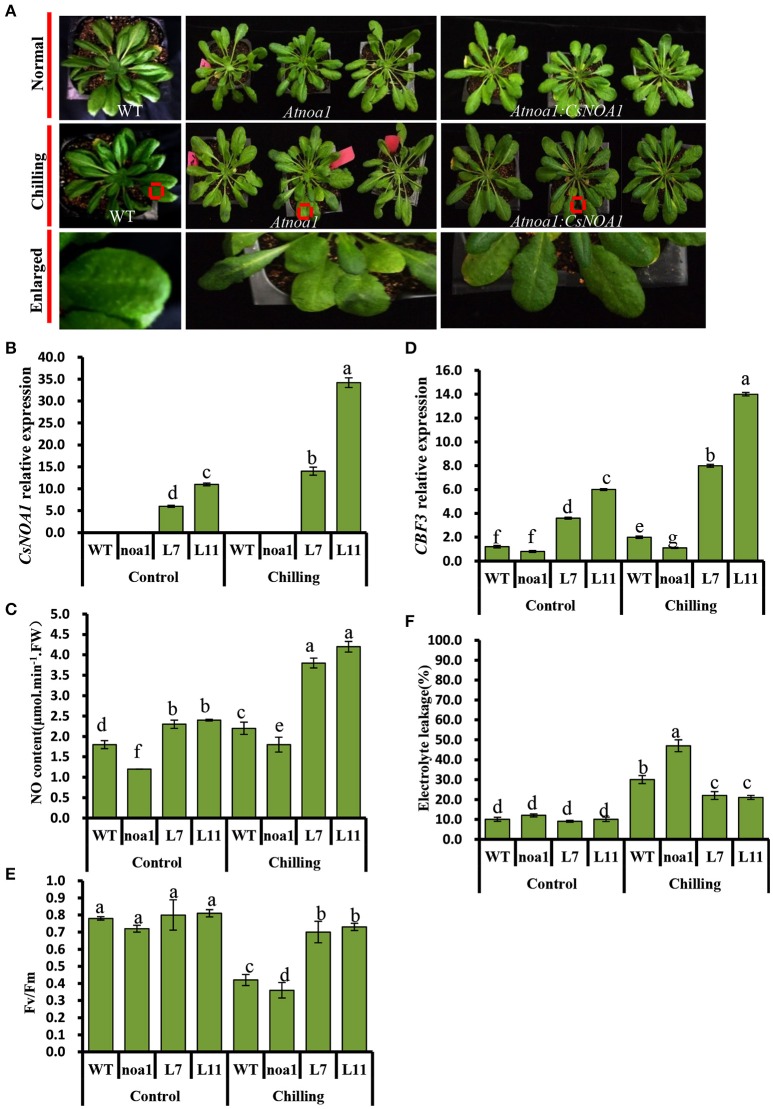
**Over-expression of *CsNOA1* in *Arabidopsis thaliana* confers tolerance of chilling stress**. **(A)** Phenotypes of transgenic *Arabidopsis thaliana* lines subjected to chilling stress. **(B)** *CsNOA1* expression levels in wild type (WT), mutant and transgenic lines under normal and chilling conditions. **(C)** Endogenous NO (nitric oxide) content under normal and chilling conditions. **(D)** *CBF3* expression under different temperature conditions. **(E)** Effect of chilling treatment on Fv/Fm in WT, mutant and transgenic *Arabidopsis thaliana* lines. **(F)** Effect of chilling treatment on electrolyte leakage in *A. thaliana*. L7 and L11 are two independent *CsNOA1* overexpression lines. Normal growth condition, 25°C, chilling treatment, 4°Cfor 10-days. Three independent experiments were performed and error bars indicate SD (*n* = 3). Different letters above the column indicate significant difference at (*P* < 0.05).

Seven-week-old T2 lines L7 and L11, as well as WT and *Atnoa1* mutant plants, were exposed to chilling conditions (4°C) for 10 days. No obvious phenotypic differences were observed between the WT and transgenic plants; however, the leaves from mutant plants grown under chilling conditions showed hydrophanous spots and wilting (Figure 5A). A quantitative assay further showed that endogenous NO levels in leaves of the transgenic *A. thaliana* lines were higher than in those of the WT and the mutant plants (Figure 5C). *CsNOA1* expression in the L11 line was more than 3.5 fold higher than that of the L7 line (Figure 5B), and the transcript levels of the cold tolerance marker gene, *CBF3*, were higher in the L11 line than in the L7 line (Figure 5D). Moreover, in both the L11 and L7 lines, a higher Fv/Fm value was observed in plants under chilling stress compared to WT plants and the *Atnoa1*mutant, even though this was still lower than in the control (Figure 5E). Electrolyte leakage under normal and chilling stress conditions was also tested, and was observed to increase significantly, with the *Atnoa1* mutant lines showing exacerbated damage under chilling stress (Figure 5F). While the chilled WT plants showed an almost 2 fold increase in leakage, this value was lower in the over-expressing (OE) plants and was more than 3-fold higher in the mutant lines. Taken together, these results indicated that over-expression of *CsNOA1* in *A. thaliana* enhanced chilling stress tolerance.

### *CsNOA1* is involved in chilling stress tolerance in cucumber

**Figure 6:**
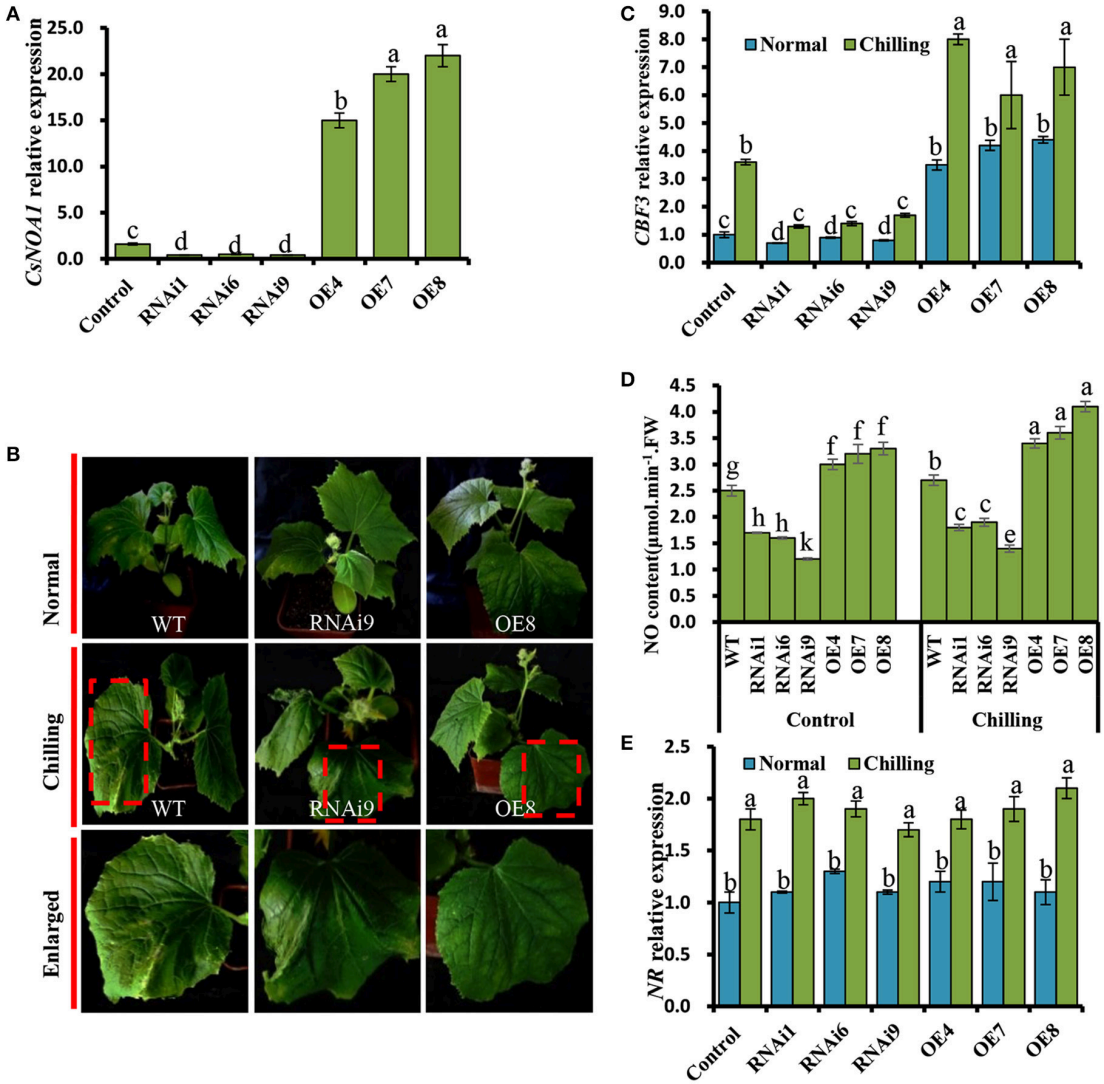
**Chilling tolerance of *CsNOA1*-OE and *CsNOA1*-RNAi cucumber lines**. **(A)** *CsNOA1* expression analysis in wild-type (WT) plants, transgenic overexpressing (OE) and RNAi lines. **(B)** Phenotypes of WT, OE, and RNAi plants at the same growth stage under normal and chilling stress conditions. **(C)** *CBF3* expression profiles under different temperature conditions in WT, OE, and RNAi lines. **(D)** The effect of chilling stress on NO content in WT, OE, and RNAi lines. **(E)** *NR* expression profile in WT and transgenic cucumber lines under different growth conditions. Normal growth condition, 25°C, chilling treatment, 4°C for 72 h. Three independent experiments were performed and error bars indicate SD (*n* = 3). Different letters above the column indicate significant difference at (*P* < 0.05).

To verify that *CsNOA1* indeed confers chilling stress tolerance, several overexpressing and knockdown cucumber lines were generated. These were evaluated by real-time quantitative PCR, and *CsNOA1* transcript levels in the knockdown lines (RNAi1, RNAi6, and RNAi9) were shown to be reduced by approximately 70% compared to WT, while *CsNOA1* transcript levels in the OE lines were more than 15-fold higher than in WT plants (Figure 6A). Seedlings of both OE and RNAi lines were phenotypically similar to WT plants under normal conditions; however, after chilling at 4°C for 72 h, the WT plants displayed symptoms such as mildly wilted leaves (Figure 6B), and this phenotype was greater in the RNAi lines. In contrast, no such stress symptoms were observed in the OE lines following the identical chilling treatment (Figure 6B). A chilling damage index (CI) was also used to evaluate the stress tolerance of the transgenic lines. As shown in Table 1, the CI of the OE lines was substantially lower than that of the WT plants, while values were highest in the RNAi lines. The chilling tolerance of the OE lines was therefore greater than that of the RNAi lines, which is consistent with *CsNOA1* playing a role in low temperature stress tolerance.

**Table 1:** **Chilling damage index (CI) in wild type and transgenic lines affected by chilling stress**.

**Line**	**Normal condition**	**Chilling condition**
Control	0	0.43 ± 0.01c
RNAi1	0	0.54 ± 0.04a
RNAi6	0	0.57 ± 0.02a
RNAi9	0	0.59 ± 0.02a
OE4	0	0.21 ± 0.04b
OE7	0	0.24 ± 0.06b
OE8	0	0.22 ± 0.04b

*Different letters in the third column indicate significant difference at P < 0.05*.

We further determined that the expression of *CBF3*, a gene that has been shown to contribute to enhancing chilling stress tolerance (Miura et al., 2007; Liu et al., 2010), was significantly up-regulated in the OE lines (OE4, OE7, and OE8), while its expression was greatly reduced in the RNAi lines (Figure 6C).

Next, a quantitative assay was performed to detect endogenous NO in leaves. We observed that the NO content of OE line leaves was higher than that of in WT and RNAi plants grown under normal condition (Figure 6D). Under chilling stress condition, the NO levels in all test plants was higher than that of in WT, OE, and RNAi plants grown under normal condition. For example, the NO content of OE8 was 1.5 times higher than that of the WT under chilling conditions, suggesting that *CsNOA1* contributed to the production of endogenous NO (Figure 6D). When the expression level of *NR* was analyzed by real time RT-PCR, no obvious change was observed between the transgenic cucumber lines and WT; however, *NR* transcript levels were generally higher under chilling conditions than under normal conditions (Figure 6E). We infer from these results that *CsNOA1* produced NO independent of *NR* expression.

### Chloroplast ultrastructure was altered in transgenic cucumber leaves

**Figure 7:**
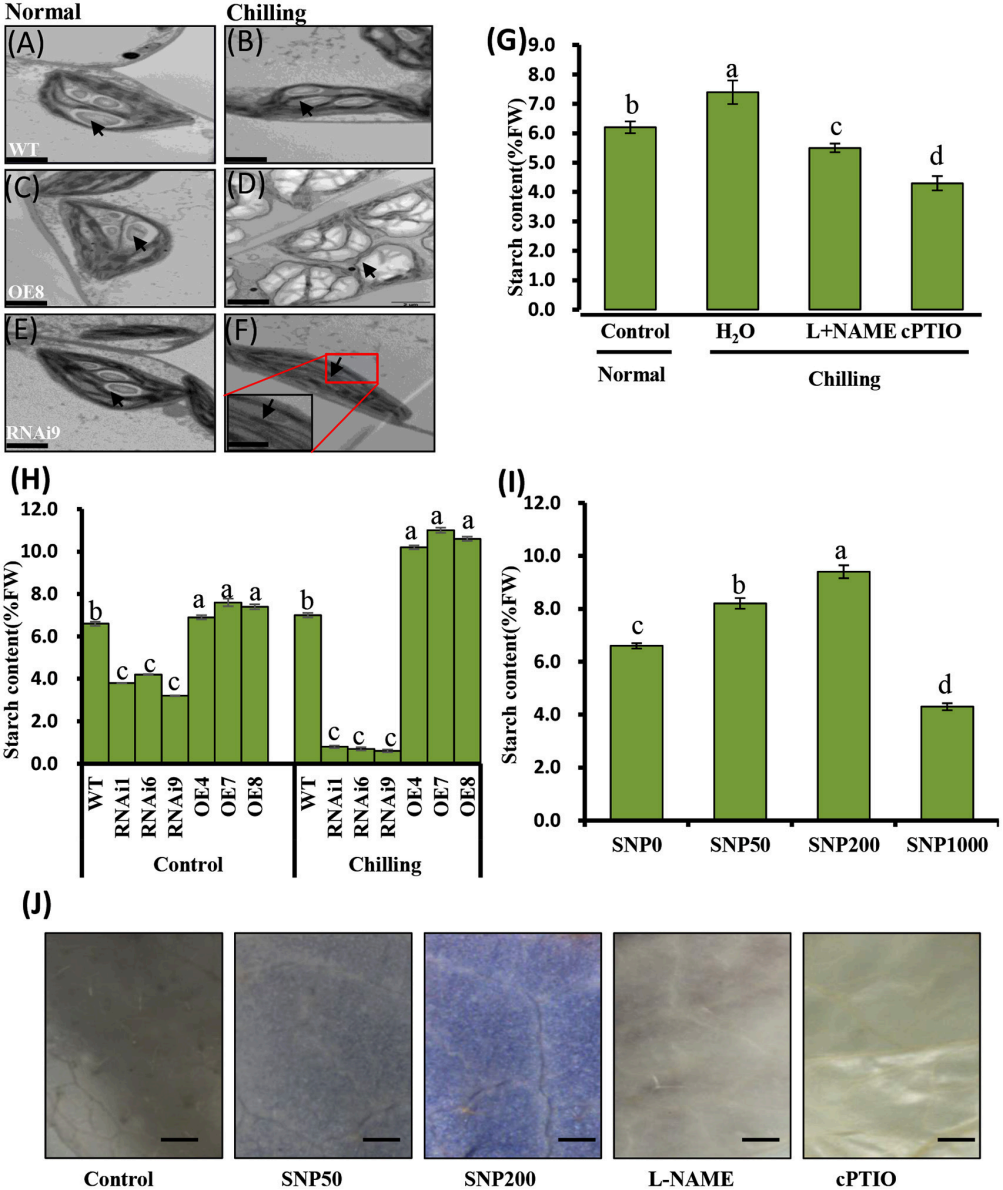
**Analyses of chilling induced ultrastructure of chloroplasts and starch content in wild type (WT) and *CsNOA1* transgenic cucumber lines**. **(A-F)** Transmission electron microscopy (TEM) images of the ultrastructural changes of chloroplasts during normal and chilling stress conditions. The black arrows indicates starch granules. Scale bar = 2 μm. **(G)** Starch content in WT cucumber leaves under chilling stress at 4°C for 2 days with or without treatment with 200 μM L-NAME, 400 μM cPTIO. **(H)** Starch content changes in WT and transgenic cucumber lines under normal and chilling conditions. **(I)** Changes of starch content in WT leaves treated with varying concentrations (0, 50, 200, and1000 μM) of SNP for 2 days at 25°C. **(J)** Cucumber leaves stained for the presence of starch with potassium iodide under different SNP concentrations. Scale bar = 2 mm. Experiments were repeated three times. Mean values of three independent samples are shown and the error bars indicate the standard errors. Different letters above the column indicate significant differences at *P* < 0.05.

**Figure 8:**
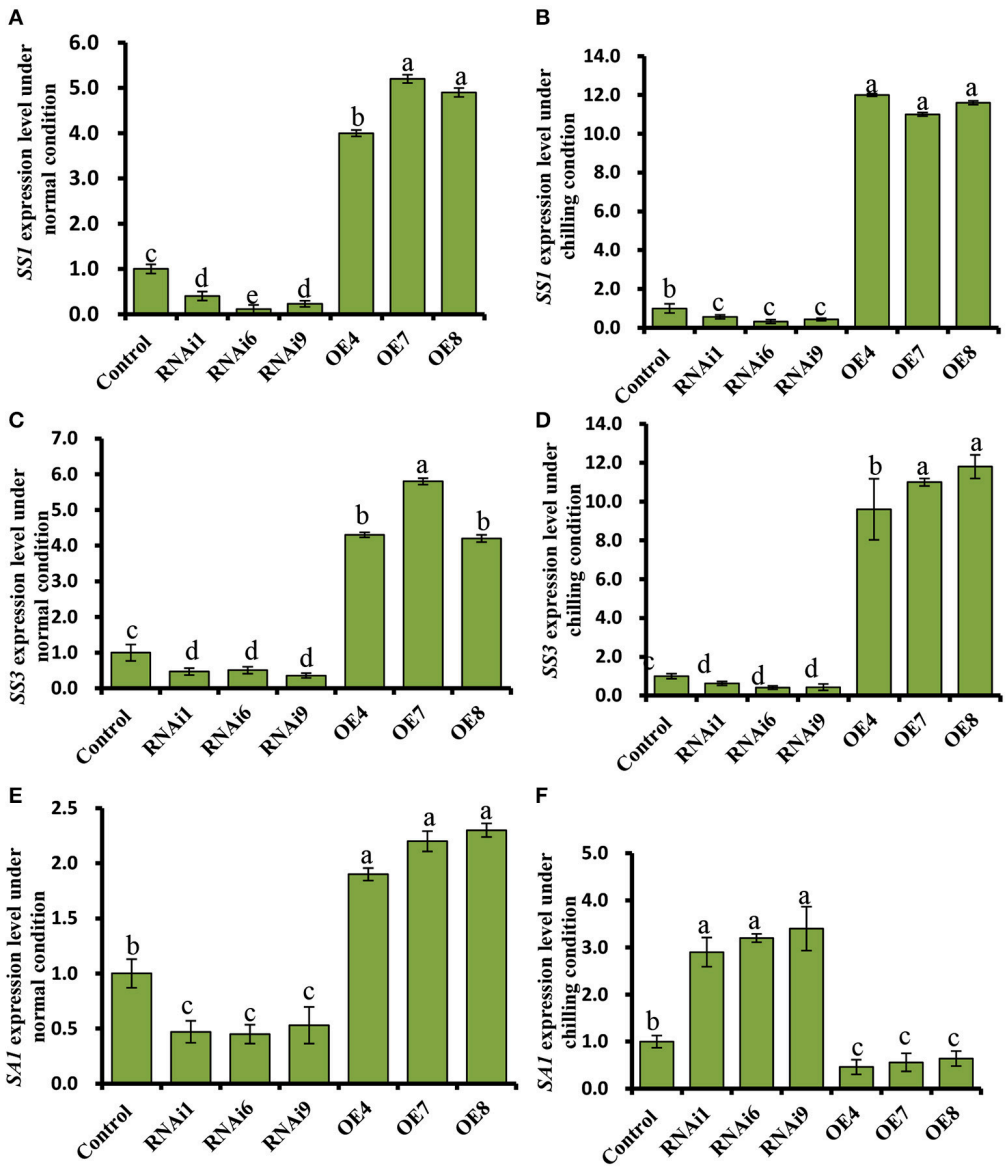
**Expression profiles of the starch synthesis related genes, *SS1*, and *SS3*, and the starch degradation related gene, *SA1*, in cucumber leaves**. **(A**,**C,E)** Expression patterns of *SS1, SS3*, and *SA1* determined by real time RT-PCR in wild type (WT) and *CsNOA1* transgenic lines grown under normal condition. **(B,D,F)** Expression patterns of *SS1, SS3*, and *SA1* determined by real time RT-PCR in WT and *CsNOA1* transgenic lines grown under chilled condition. The cucumber α*-TUBULIN (TUA)* gene was used as an internal control. The experiments were repeated three times and the mean values of three independent samples and standard errors are presented. Different letters above the column indicate significant differences at *P* < 0.05.

WT cucumber leaves had smaller shrunken chloroplasts when subjected to chilling stress, unlike non-stressed leaves (Figures 7A,B). The chloroplasts in the OE8 line appeared rounded, with large starch granules, in contrast to those of the WT under the same conditions (Figures 7A–D), while small starch granules were observed in the chloroplasts of the RNAi3 leaves (Figures 7E,F). Starch content was measured and found to increase from 6.5 to 7.3% of fresh weight in WT leaves after 4 days of chilling at 4°C (Figure 7H). Incubation under the same conditions led to an increase of starch content in the OE8 line from 7.5 to 9.2% of fresh weight, while a dramatically decrease was observed in the RNAi9 line (Figure 7H). Given that the NO level was lower in the RNAi lines than in the WT plants, both under non-chilling stress (control) and chilling stress conditions (Figure 6D), we hypothesized that the accumulation of starch may be regulated by endogenous NO. To confirm this, we investigated the relationship between endogenous NO levels and starch content in the leaves of WT plants grown under chilling conditions. This study was performed by manipulating endogenous NO levels using an NO scavenger and inhibitor. Figures 7G,I show that low concentration (<1000 μM) exogenous nitric oxide donor SNP stimulated starch accumulation under normal conditions, while the increased starch content induced by chilling was markedly inhibited by treatment with cPTIO and L-NAME. A higher starch accumulation in leaves with treatment of SNP and its inhibitor, was evident by a darker color when stained with potassium iodide (Figure 7J). These results indicate that endogenous NO may function as a trigger to promote starch accumulation as a consequence of chilling stress. We used real time RT-PCR to analyze the expression levels of the starch synthesis related genes *SS1* and *SS3*, as well as that of *SA1*, which is related to starch degradation. In Figures 8A,C, the expression of the *SS1* and *SS3* genes in OE lines were higher than in WT and the RNAi lines under normal conditions; however, chilling resulted in an obvious increase in *SS1* and *SS3* expression in OE lines (Figures 8B,D). The expression of the starch degradation related gene, *SA1*, was also higher in WT than in the RNAi lines under normal conditions; however, chilling resulted in a decrease in *SA1* expression in WT plants (Figures 8E,F), while expression increased in the RNAi lines under chilling stress. These results indicate that an up-regulation of *SS1* and *SS3* and a down-regulation of *SA1* expression may confer greater starch accumulation in the OE lines and WT under chilling stress.

### *CsNOA1*-overexpressing plants accumulate high levels of soluble sugars, but not of proline

**Figure 9:**
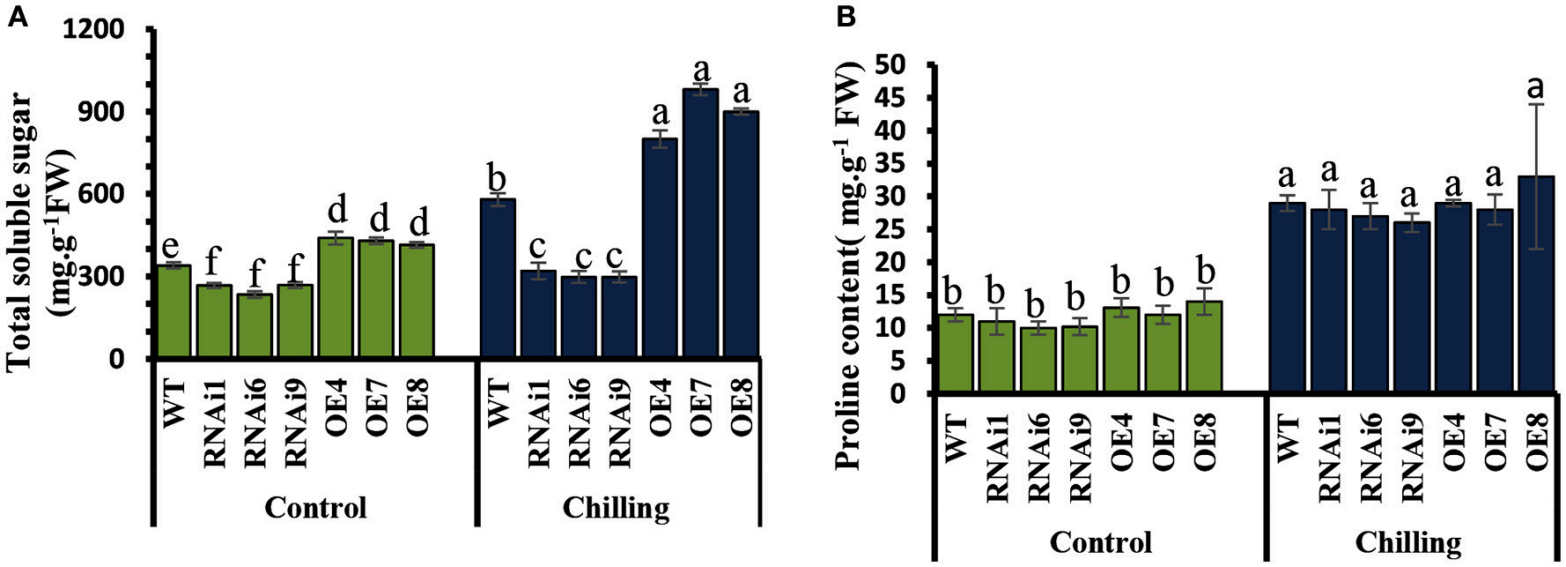
**Effect of chilling stress on levels of soluble sugars and proline in WT and transgenic cucumber lines. (A)** Comparison of soluble sugar contents in leaves from wild-type (WT), *CsNOA1* OE (over-expressing), and *CsNOA1* RNAi lines. **(B)** Changes in proline content in leaves from WT, OE, and RNAi lines. Error bars indicate the standard errors and different letters above the column indicate significant differences at *P* < 0.05.

Under normal conditions, the levels of soluble sugars in the transgenic lines showed no significant difference compared to WT (Figure 9A), while an increase was observed upon exposure to chilling stress in both WT and transgenic plants. However, the increase in soluble sugars in the *CsNOA1*-overexpressing plants was substantially higher than in WT plants. No significant difference in proline content was observed between WT and transgenic lines both under normal and chilling conditions (Figure 9B). The expression of genes associated with proline biosynthesis (*CsP5CS1*) and proline transport (*CsProT1*) was investigated and, as shown in Supplementary Figures S2A,B, after exposure to chilling stress, there was no significant difference in their transcript levels among the OE lines, the WT and the RNAi lines. We therefore concluded that *CsNOA1* affects soluble sugar accumulation but not proline levels.

### Two independent pathways NOA1- and NR- may generate endogenous NO in cucumber leaves

**Figure 10:**
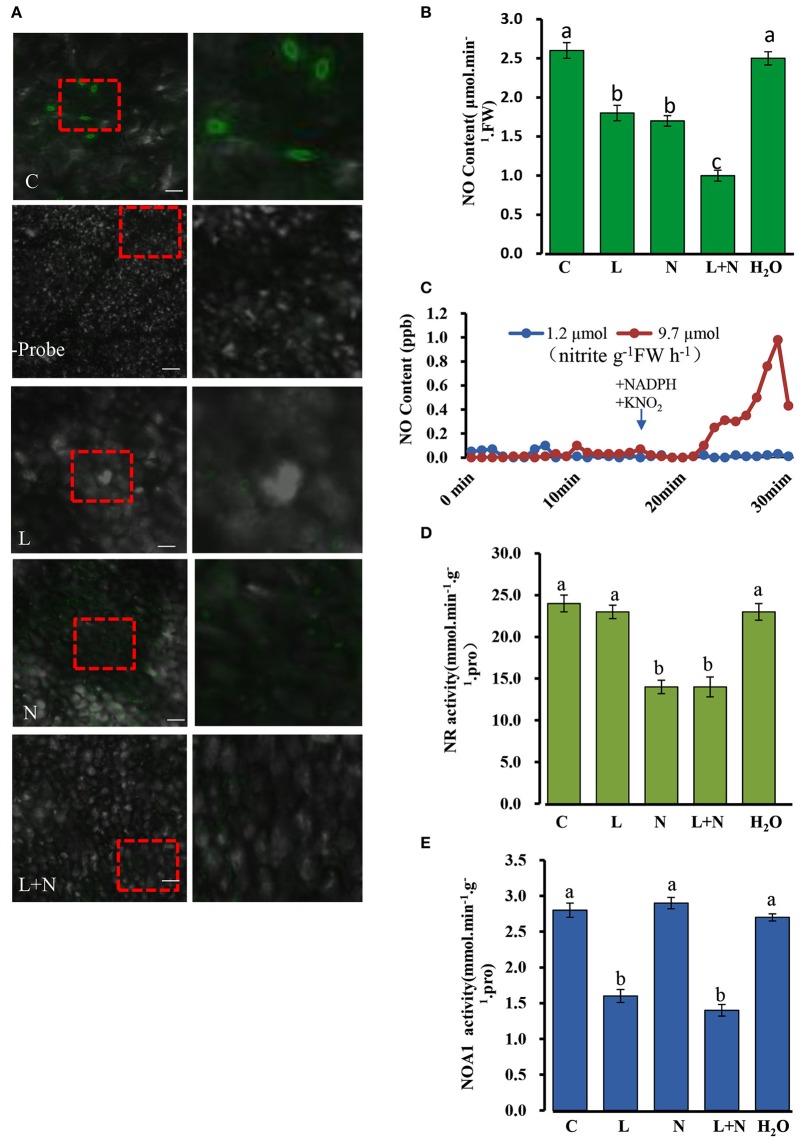
**Changes in endogenous NO (nitric oxide) levels in cucumber stomata under normal conditions**. **(A)** NO levels in excised leaves were monitored by labeling with the NO-specific fluorescent probe DAF-FM DA and imaged by confocal microscopy. The right-hand image is a magnification of the red dotted square in the left image. Scale bar = 100 μm. Two hundred micrometer NaN_3_ and 200 μM L-NAME were applied to wild type cucumber plants grown under normal conditions. **(B)** NO levels in cucumber leaves under normal conditions. **(C)** NR involved in the NO production from nitrite plus NADH. Two ml of desalted cucumber extract were pre-incubated for 17 min with 5 mM AMP and 20 mM EDTA (open symbols), or with 5 mM ATP and 50 μM cantharidin (closed symbols) in a Petri dish placed in a reaction chamber (3.01–3.02 air min^−1^, room temperature); NADH (250 μM) and KNO_2_ (100 μM) were added as indicated. **(D,E)** Effect of inhibitors on NOA1 and NR activity under normal conditions. L, L-NAME; N, NaN_3_; C, wild type and H_2_O was used as a control. The mean values of three independent samples and standard errors are shown, and the same letter above the column indicates no significant differences at *P* < 0.05.

In order to determine whether endogenous NO is present in cucumber seedlings, we used the fluorochrome DAF-2DA to detect NO at the three leaf stage. Figure 10 shows that under confocal laser scanning microscopy (CLSM) an intense green fluorescence was present in the leaf stomata in fluorochrome treated samples, but no fluorescence was observed when the fluorescent probe was omitted. Likewise, NO-derived fluorescence was detected in cucumber leaf sections pre-incubated with the NOS1/NOA1 activity inhibitor, L-NAME, and we observed that the green fluorescent spots were not brighter than those in the control samples. As with the L-NAME treatment, green fluorescent spots were also observed in cucumber leaf sections pre-incubated with the NR activity inhibitor, sodium azide (NaN_3_) (Figure 10A). Interestingly, NO-derived fluorescence was barely detectable in leaf sections pre-incubated with both L-NAME and NaN_3_.

A quantitative assay was performed to detect endogenous NO levels (Figure 10B). Treatments with the inhibitors, L-NAME or NaN_3_, significantly reduced NO levels in all leaf samples compared to those of the control, and when the two inhibitors were used in combination, endogenous NO levels decreased even more substantially, although they were not abolished entirely. In addition, we repeated the experiments using the inhibitors L-NNA (Rockel et al., 2002) for NOA1 and tungstate (Planchet et al., 2005) for NR, which gave similar results to those described above (Supplementary Figure S3A–D). Taken together these results suggest that NOA1 and NR act primarily in the endogenous NO pathway.

It is known that NO production can be modulated by preincubation with ATP, which indicates that NR activity (Rockel et al., 2002; Planchet et al., 2005). Desalted cucumber leaf extracts was used instead of purified NR in this study. It has been reported that auxiliary enzymes (NR kinase, P-NR phosphatase and 14-3-3 proteins) are required for this experiment (Rockel et al., 2002). Following the addition of NADH plus nitrite in 2 ml of reaction medium, NO was emitted into the gas phase above the solution. We observed that approximately 10 min after substrate addition, NO emission decreased again. In contrast, pre-incubation of the extract with ATP, Mg^2+^ and the protein phosphatase 2A inhibitor, cantharidine, caused an almost complete inhibition of nitrite-dependent NO production. The measured NR activity in an aliquot of the extract was 9.7 μmol nitrite g^−1^FW h^−1^ for the AMP pretreated samples, but 1.2 μmol nitrite g^−1^FW h^−1^ after pre-incubation with ATP plus cantharidine (Figure 10C). This result suggested that NR is involved in NO production in cucumber leaves.

NR and NOA1 activities were also measured in the presence of other inhibitors, and NOA1 activity was markedly reduced after adding its inhibitor L-NAME, but was not affected by NaN_3_ (Figures 10D,E). Similarly, NR activity declined substantially after adding its inhibitor NaN_3_, but was not affected by L-NAME. These results suggest that generation of endogenous NO in cucumber leaves may occurs largely independently in the NOA1 and NR pathways.

## Discussion

Cucumber is a warm-season horticultural crop with little, or no, frost tolerance (Liu et al., 2010), and while it is known that low temperature stress can be alleviated by NO in plants such as maize, rice and *A. thaliana* (Zhao et al., 2009; Liu et al., 2011; Wimalasekera et al., 2011; Yang et al., 2011b; Tan et al., 2013b), the molecular regulation of chilling tolerance by NO in cucumber is largely unknown.

### Mitochondria localized CsNOA1 confers chilling stress tolerance in transgenic *A. thaliana*

Previous studies showed that NOA1 activity is linked to peroxisomes and chloroplasts (Corpas et al., 2001; Gould et al., 2003), while only a few reports describe mitochondria localized NO synthesis (Guo and Crawford, 2005; Hao et al., 2010). However, the Arabidopsis AtNOS1 gene or NOA1 gene, are not yet accepted as the genes involved in NO synthesis in plants. Initially AtNOS1 was considered as plant NOS (Guo et al., 2003) and is yet to be proven. Therefore, these disputed conclusions are not to be interpreted to reflect a standard NOS present in plants. In this current study, GFP-fused CsNOA1 was observed in the mitochondria, as fluorescence overlapped with Mito-tracker Red in the stomata, but not with Chl fluorescence (Figure 4). In further support of CsNOA1being localized to the mitochondria, we also observed that CsNOA1-GFP fluorescence overlapped with Mito-tracker Red in roots (Supplementary Figure S4).

We observed that ectopic expression of *CsNOA1* in the *A. thaliana* mutant *noa1*rescued the mutant phenotype and enhanced its tolerance to chilling stress (Figures 5A–F), which may be a consequence of increased levels of NO. Such an association with NO signaling has also observed in other plants. For example, studies using NR inhibitors, a NO scavenger, and a NO donor, showed that *NR*-dependent NO levels are positively correlated with freezing tolerance (Zhao et al., 2009). We observed that the degree of tolerance correlated with the level of *CsNOA1* expression (Figure 5), with a strong over-expression of *CsNOA1* resulting in a high degree of cold tolerance and high endogenous NO levels. This suggests that *CsNOA1* is important for chilling stress, and that the effect is dose dependent. We determined that *CsNOA1* mRNA levels in the L11 line were 3.5 fold higher than those in the L7 line, and that the chilling index (CI) and the expression of the cold tolerance marker gene *CBF3* was lower (Figure 5D). We also saw a higher Fv/Fm ratio in plants subjected to chilling stress, even though it was still lower than in plants grown under normal condition (Figure 5E).

### Transgenic cucumber *CsNOA1* overexpressing and RNAi lines exhibited altered chloroplast ultrastructure and *CBF3* expression patterns

Phenotypic observation and physiological indicators, such as Fv/Fm and *CBF3* expression, suggested a greater chilling tolerance in lines *CsNOA1* OE lines than in the *CsNOA1* RNAi lines (Figure 6). *NOA1* is required for chloroplast biogenesis (Flores-Pérez et al., 2008), and the ultrastructure of chloroplasts was therefore observed in all the different lines (Figures 7A–D). The chloroplasts of the OE lines were shown to be filled with swollen starch granules, unlike those of the non-transgenic control grown under the same conditions (Figure 7A), and we propose that an important function of *NOA1* is to ensure efficient chloroplast function during photosynthesis.

A major response of plants to chilling is the activation of the *CBF*-dependent pathway, which regulates the CBF regulon (Van Buskirk and Thomashow, 2006). In this study, we showed that the expression of *CBF3* was greatly induced in chilled *CsNOA1* OE lines compared with WT plants. In contrast, the level of *CBF3* expression was lower in the RNAi lines compared with WT plants under the same chilling stress conditions (Figure 6). These results indicate that *CsNOA1* affected the expression of *CBF3*, and link NO signaling with the *CBF*-dependent pathway. This is supported by a previous report suggesting that in the *nia1nia2* NR double mutant, NO production is linked to the *CBF*-dependent pathway (Cantrel et al., 2011). We suggest that under chilling conditions, different sources of NO in different plants may be involved in the same cold/chilling signal pathway, or at least, in the *CBF*-dependent pathway.

### *CsNOA1* affects soluble sugar accumulation, but not proline levels, in chill stressed transgenic cucumber lines

We observed that elevated NO levels can result in starch accumulation in cucumber leaves under normal conditions (Figures 7I,J), and that under chilling conditions, the starch content in *CsNOA1* OE cucumber lines was higher than in WT plants (Figures 8A–D). Therefore, the induction of starch accumulation may be a downstream effect of *CsNOA1*-induced NO production. To date, there have been few reports showing that NO can alter the expression of the starch biosynthesis related genes *SS1* and *SS3* or the starch degradation related gene *SA1* (Figures 8A–F, Supplementary Tables 1–2). Our study provides evidence that NO signaling is linked to starch content (Supplementary Tables 1–2) and we detected an enhanced accumulation osf soluble sugars in the *CsNOA1* OE lines, which may partially account for the higher tolerance of these plants to chilling stress. The high transcript level of the *SUG* gene, which encodes an alkaline/neutral invertase, has previously been correlated with glucose accumulation (Maruyama et al., 2014), and a similar relationship between high transcript levels of this gene and enhanced levels of glucose has been reported in cold-treated *A. thaliana* plants (Kaplan et al., 2007).

*SUG* expression in the *CsNOA1* OE lines, RNAi lines, and WT cucumber seedlings in response to chilling stress were more than 6-fold higher in the OE lines in untreated plants (Supplementary Figure 2C), while the other three *SUG* genes showed no changes in all tested samples (data not shown). High expression of the *SUG* gene in the *CsNOA1* OE lines may effectively enhance osmoregulation capacity and improve water potential by the accumulation of sugars, thereby minimizing chilling induced damage.

Land plants accumulate free proline in response to a number of abiotic stresses, such as drought, salinity and freezing (Hare et al., 1999; Ashraf and Foolad, 2007; Hoque et al., 2007). We detected no changes in proline content in cucumber *CsNOA1* OE or RNAi lines when compared to WT before or after chilling stress (Figure 9B), as well as no differences in the expression of proline biosynthesis or transport related genes (Supplementary Figure S2A,B). In contrast, total soluble sugar levels increased substantially in the OE lines and were greatly reduced in the RNAi lines after exposure to chilling stress, when compared to WT plants (Figure 9A). This result is not congruent with an analysis of cold-stressed *A. thaliana* (Zhao et al., 2009), suggesting that the ameliorative effect of NO on chilling stress tolerance cannot be explained by osmoregulation mediated by proline content. It may also be that NO is not directly related to proline content or P5CS activity, and that the expression of *P5CS* is not directly regulated by NO.

### NOA1 and NR, this two pathways co-exist in cucumber seedlings

**Figure 11:**
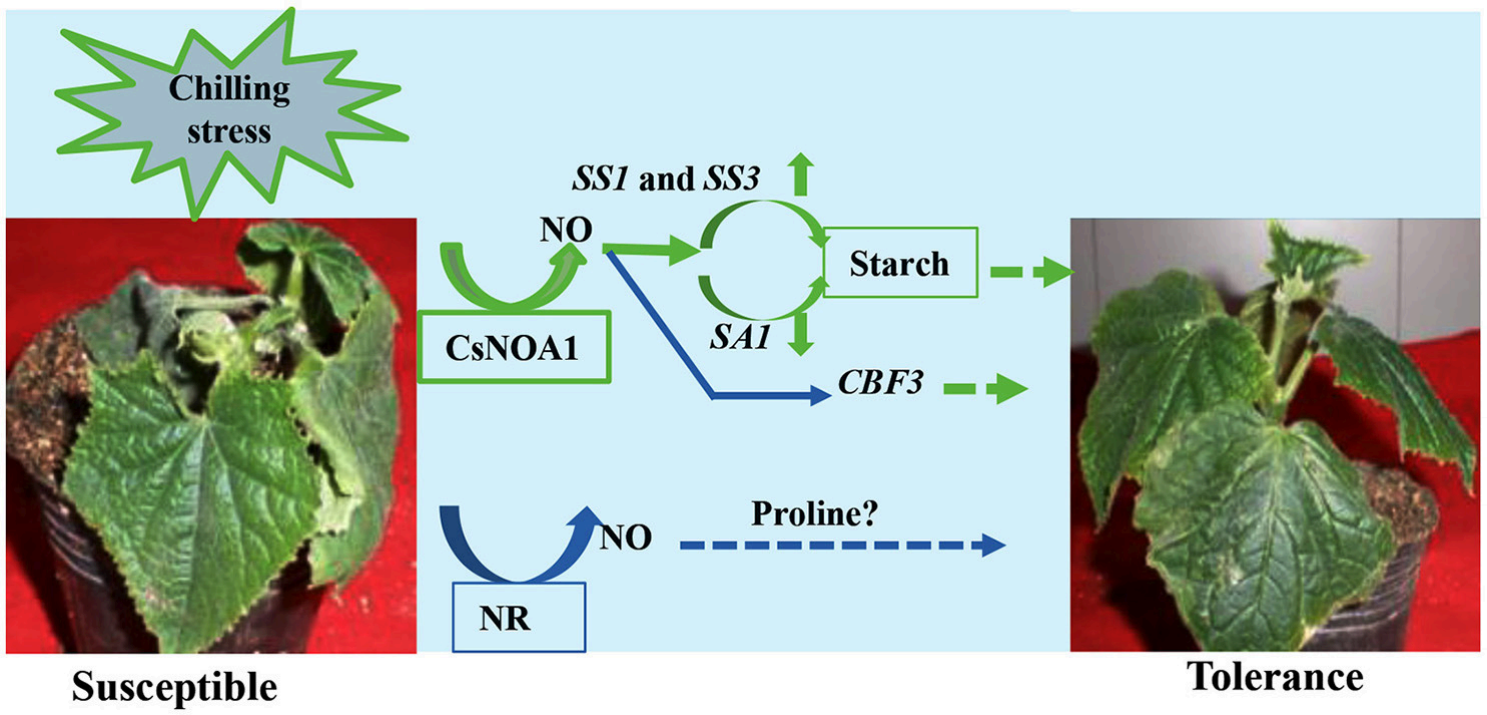
**A Putative working model of the involvement of *CsNOA1* in chilling stress**. NO (nitric oxide) produced by the *CsNOA1* pathway activates the starch metabolic pathway, leading to an increase in starch content. *CBF3* expression is also affected by NO. *CBF3*-denpendent stress tolerance may be correlated with, or independent of, starch metabolism. Endogenous NO is generated independently of the NR pathway in cucumber.

Under normal condition, we took several classic inhibitors (Zhao et al., 2007a, 2009; Rümer et al., 2009) to perform and detect endogenous NO levels in wild type cucumber seedling (Figure 10; Supplementary Figures 3A–D). The results suggest that generation of endogenous NO in cucumber leaves occurs largely independently in the NOA1 and NR pathways. Under chilling condition, when the expression level of *NR* was analyzed by real time RT-PCR, no obvious change was observed between the transgenic cucumber lines and WT; however, *NR* transcript levels were generally higher under chilling conditions than under normal conditions (Figure 6E). We infer from these results that *CsNOA1* produced NO independent of *NR* expression This result is not consistent with studies of loquat fruit (Xu et al., 2012), where chilling induced NO generation was partially suppressed by the NR inhibitor tungstate, indicating that the NR pathway may be the main source of NO generation. Additionally, in *A. thaliana*, endogenous NO production is mainly generated by NR activity in WT leaves and chilling-induced NO release is reduced in the *NR* double mutant (Zhao et al., 2009; Cantrel et al., 2011). The results indicate that in cucumber, the two pathways can operate together under nomal condition, but under chilling stress, this stress signal, may trigger a certain pathway to produce NO and confer its tolerance to stress. Because of the lack of NOA1-and NR- related mutants in cucumber, inhibitors were chosen as a study tool, although those provide an indirect confirmation. Using new technology, for example, CRISPY-Cas9, to create CsNOA1 and NR null mutants would further verify this result. However, this new technology was technically immature in cucumber, and presently work is being performed in this area. Considering all the data, a model (Figure 11) is proposed in which *CsNOA1* transcription occurs independent of chilling stress. NO produced by CsNOA1 directly activates the expression of *CBF3* and, meanwhile, NO activates the starch metabolic pathway, leading to increased expression of *SS1* and *SS3* and decreased expression of *SA1*. High expression of *CBF3* and accumulation of starch increases the stress tolerance of cucumber.

### Accession numbers

Sequence data of NOA1 proteins in this study can be found in the cucumber Genome database, Arabidopsis Genome Initiative, Phytozome or Genbank/EMBL/Swiss-prot database and NCBI under the following accession numbers: *CsNOA1* (Csa5M168870), *Atnoa1* (At3G47450), *Zea mays* (NP-001168044.1), *Oryza sativa* (Os02g0104700), *Brassica juncea* (Bradi3g00690.1), *Nicotiana benthamiana* (AB303300.1), *Ricinus communis* (XP002510962.1), *Medicago truncatula* (KEH36486.1), *Solanum tuberosum* (GU205181.1). For real-time PCR, sequence data of text genes in this study can be found in the cucumber Genome database under the following accession numbers: *CsP5CS1* (Csa3M073290), *CsSug1* (Csa7M308910), *CsSug2* (Csa2M034660), *CsSug3* (Csa3M168930), *CsSug4* (Csa5M615240), *CsCBF3* (Csa5M155570), *CsSS1* (Csa6M497160), *CsProtrans* (Csa5M615830), *CsSS3* (Csa1M062920).

## Author contributions

HR and XL designed the experiments and wrote the first draft and generated Figures 1–11. BL, and Shudan Xue contributed to genes expression analyses. YC, WQ, CJ, Shuo Xue and TW contributed to a second draft. All of the authors revised the manuscript multiple times. HR, XL, and BL performed the final revision of the manuscript, which was read and approved by all authors.

### Conflict of interest statement

The authors declare that the research was conducted in the absence of any commercial or financial relationships that could be construed as a potential conflict of interest.

## Abbreviations

CBF3: C-repeat binding factor 3
GFP: green fluorescent protein
GUS: β-glucuronidase
NO: nitric oxide
NOA1: nitric oxide associated1
NR: nitrate reductase
OE: overexpressing
RNAi: RNA interference
WT: wild type.
