# Supplementary material for: Cucumber (Cucumis sativus L.) Nitric Oxide Synthase Associated Gene1 (CsNOA1) Plays a Role in Chilling Stress
Source: Front Plant Sci. 2016 Nov 11;7:1652. doi: 10.3389/fpls.2016.01652 (PMC5104743; doi:10.3389/fpls.2016.01652)
Supplement: Supplementary file 1 [file Table1.DOCX]

**Supplementary Table1**. **List of starch related genes that up-regulated in *CsNOA1* overexpression plants**

|  |  |  |
| --- | --- | --- |
| Accession | Description | Ratio(OE/WT) |
| Csa1M062920 | Starch synthase III | 4.2 |
| Csa3M751970 | Starch branching enzyme I | 4.2 |
| Csa3M825040 | Starch synthase isoform II | 4.0 |
| Csa3M889910 | Isoamylase-type starch-debranching enzyme 3 | 3.9 |
| Csa5M146950 | Granule-bound starch synthase | 3.9 |
| Csa5M599830 | Starch-granule-bound R1 protein | 3.5 |
| Csa6M497160 | Starch synthase V | 2.4 |
