# Supplementary material for: Cucumber (Cucumis sativus L.) Nitric Oxide Synthase Associated Gene1 (CsNOA1) Plays a Role in Chilling Stress
Source: Front Plant Sci. 2016 Nov 11;7:1652. doi: 10.3389/fpls.2016.01652 (PMC5104743; doi:10.3389/fpls.2016.01652)
Supplement: Supplementary file 2 [file Table2.DOCX]

**Supplementary Table2. List of starch related genes that down-regulated in *CsNOA1* RNAi plants**

| Accession | Description | Ratio(RNAi/WT) |
| --- | --- | --- |
| Csa7M21310 | Starch-branching enzyme-like protein | 0.5 |
| Csa7M21310 | Putative starch branching enzyme | 0.4 |
| Csa7M39230 | Isoamylase-type starch-debranching enzyme 2 | 0.4 |
| Csa5M58530 | Carbohydrate transporter/ sugar porter/ transporter | 0.4 |
| Csa6M51690 | Sugar transporter, putative | 0.3 |
