# Supplementary material for: Cucumber (Cucumis sativus L.) Nitric Oxide Synthase Associated Gene1 (CsNOA1) Plays a Role in Chilling Stress
Source: Front Plant Sci. 2016 Nov 11;7:1652. doi: 10.3389/fpls.2016.01652 (PMC5104743; doi:10.3389/fpls.2016.01652)
Supplement: Supplementary file 3 [file Table3.DOCX]

**Supplementary Table 3. Primes used in this study**

| Primes | Prime sequences(5’-3’) | Engineered site | Purpose |
| --- | --- | --- | --- |
| R11F | GCTCTAGA ATGGCACTCGCTCCCTTCT | XbaI | OE |
| R12R | TCCCCCGGGCCAAAGTACCATCTTGGTCTTA | SmaI |  |
| R13F | GTCGACATGGCACTCGCTCCCTTCT | SaiI | GFP |
| R14R | CCGCGGAAAGTACCATCTTGGTCTTA | KpnI |  |
| R15F | GGGATCCCATGGCACTCGCTCCCCTTCT | AscI | RNAi |
| R16R | ACTAGTGCAACGGAGCACCACAGCCAT | SwaI |  |
| R17F | GGATCCATGGCACTCGCTCCCTTCT | BamHI |  |
| R18R | GGCGCGCCGCAACGGAGCACCACAGCCAT | SpeI |  |
| R19F | GAGCTCGTGGAGGTGACAATGG |  | *NR*  qRT-PCR |
| R20R | GGTACCGGTGGATATTTCTAGATG |  |  |
| R21F | GAGAGGGGTAAACAGTGAATC |  | Actin for cucumber |
| R22R | ACGCTGTTGGTGGTGGTAC |  |  |
| R23F | AGCGATGGCTGGAACAGAAC |  | Actin For Arabidopsis |
| R24R | CCTTCGTCTTGATCTTGCGG |  |  |
| R25F | CATACAATCTGCTGTTCCTGG |  | Probe for *ISH* |
| R26R | TTGGACCATAGAATGTCAAAC |  |  |
| R27F | ACGATGTAGTTATCTGTCAG |  | *SA1*  qRT-PCR |
| R28R | GTCCTCTGCTTCTTAAACCT |  |  |
| R29F | GGGAAGATGGTGGGATATTT |  | *SS3*  qRT-PCR |
| R30R | GGGAAGATGGTGGGATATTT |  |  |

| Primes | Prime sequences(5’-3’) | Engineered site | Purpose |
| --- | --- | --- | --- |
| R31F | GCTTAGAAGAGTTATCTGC |  | *CSNOA1*  qRT-PCR |
| R32R | ACCTTAGTGACCACTAGT |  |  |
| R33F | CTATATTTTGGGGAGGTC |  | *CSNOA1*  semi-RT-PCR |
| R34R | TTCGAGTCCTCTCCAATCAT |  |  |
| R35F | GATCCAATTGGTCATGTATTG |  | *CsP5CS1*  qRT-PCR |
| R36R | GGAATGGCTTCTGTGATAAC |  |  |
| R37F | ATCTGTTCATGATTAATGTGG |  | *CsProt1*  qRT-PCR |
| R38R | TGCAGATGCTCCTATTGTCG |  |  |
| R39F | AGGTTATTACTTCCAAGAGAGAGG |  | *CsSug1*  qRT-PCR |
| R40R | CTAATCTCCCCCTCGCCGTT |  |  |
| R41R | CCGGTCGGAAAAAATTTCAAG |  | *CsCBF3*  qRT-PCR |
| R42F | TTAATCGCCATGGCTCGAAT |  |  |
| R43R | GGTATGACTCCAGAAGGTGGA |  | *CsSug*4  qRT-PCR |
| R44F | TAATAAACAATGGATTCCCG |  |  |
| R45R | TCTAGATACGAGTTATCTTTTGAG | XbaI | P*_CSNOA1_-*GUS |
| R46F | GGATCCAGGTGAACGTTGAAGTGAGC | BamHI |  |
| R47F | AGAGTTGCTCCTGTTGACTC |  | *CsSug*2  qRT-PCR |
| R48R | GGTAGCCATATACACCCATT |  |  |
| R49F | GTTTTGAATT ATGATCAGGT |  | *CsSug*3  qRT-PCR |
| R50R | TCCAGAGTCAACAGGAGCAA |  |  |
| R51F | CTGTGCGGTTTTTCCATTGC |  | *CsSS1*  qRT-PCR |
| R52R | CAACTTCCTCCCCATATGGT |  |  |
