# Supplementary figures and images for: Cucumber (Cucumis sativus L.) Nitric Oxide Synthase Associated Gene1 (CsNOA1) Plays a Role in Chilling Stress
Source: Front Plant Sci. 2016 Nov 11;7:1652. doi: 10.3389/fpls.2016.01652 (PMC5104743; doi:10.3389/fpls.2016.01652)

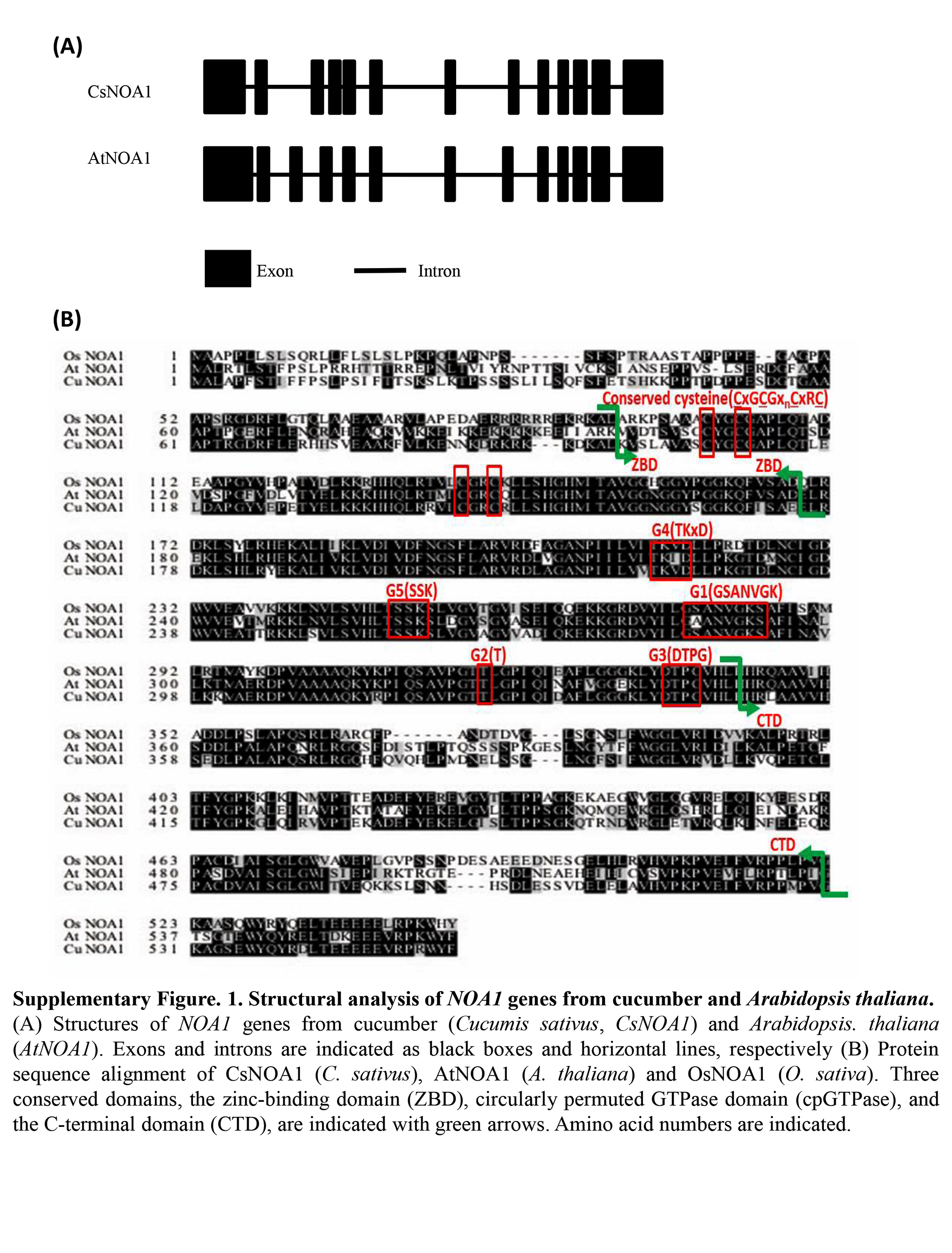

Supplement: Supplementary file 4 [file Image1.TIF]

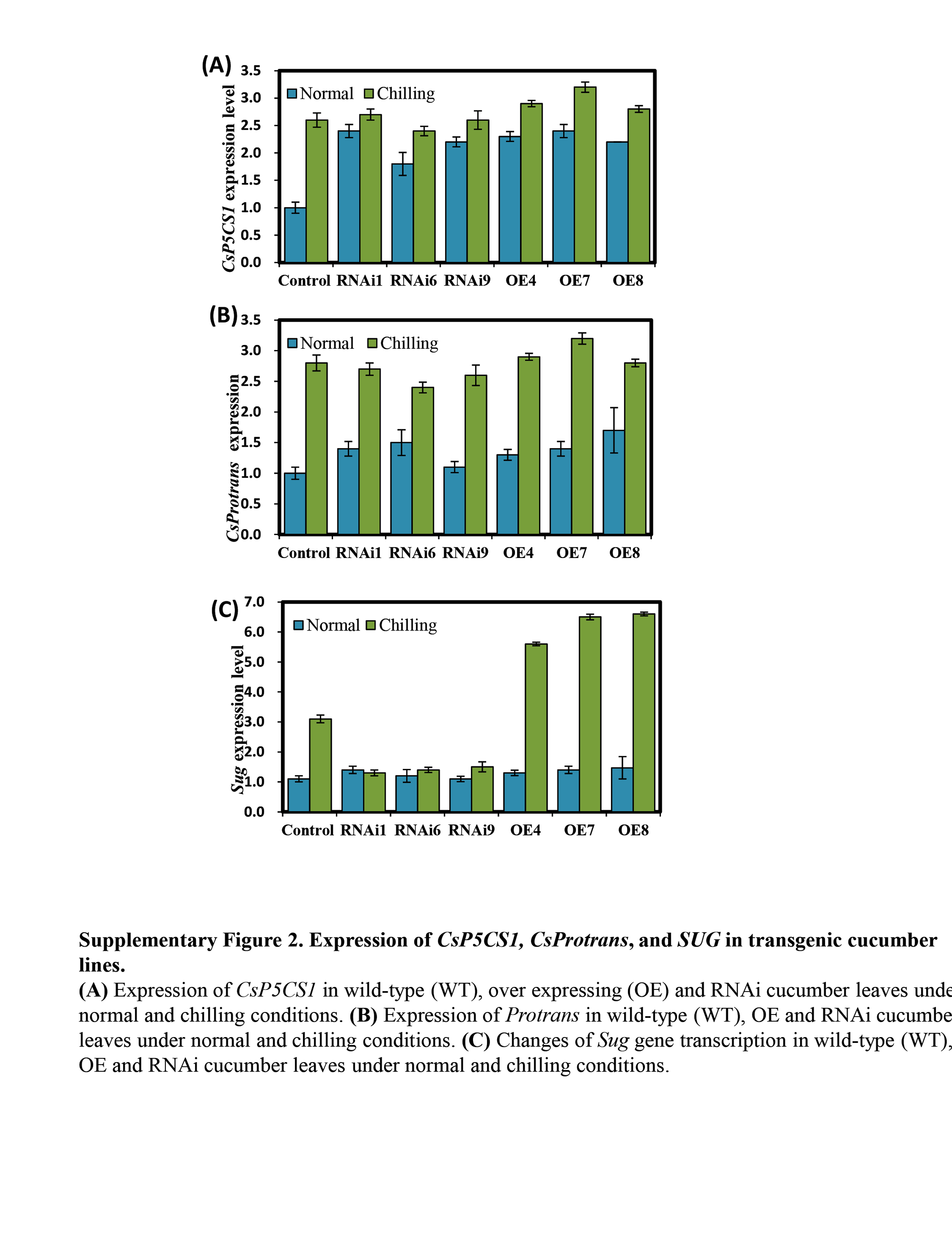

Supplement: Supplementary file 5 [file Image2.TIF]

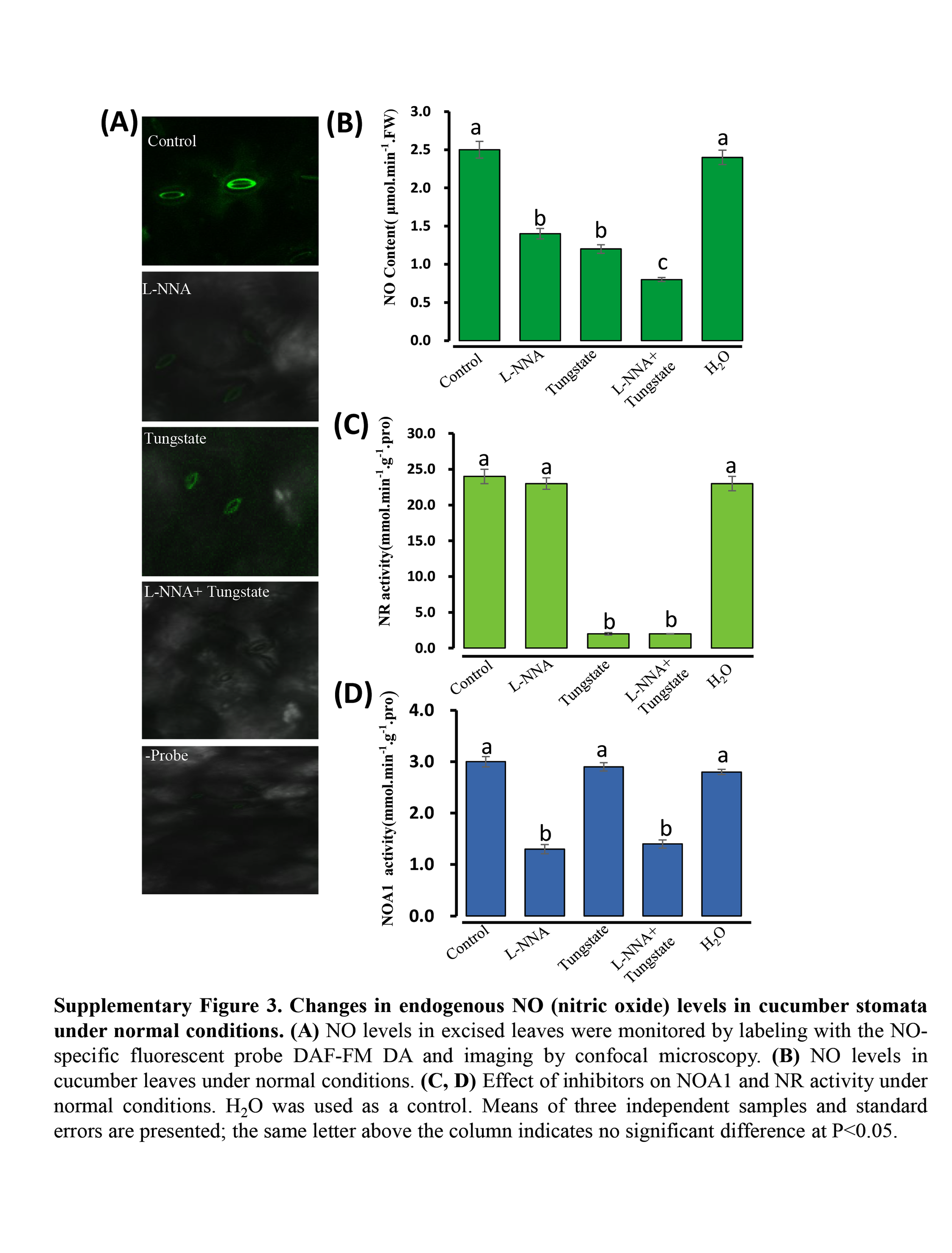

Supplement: Supplementary file 6 [file Image3.TIF]

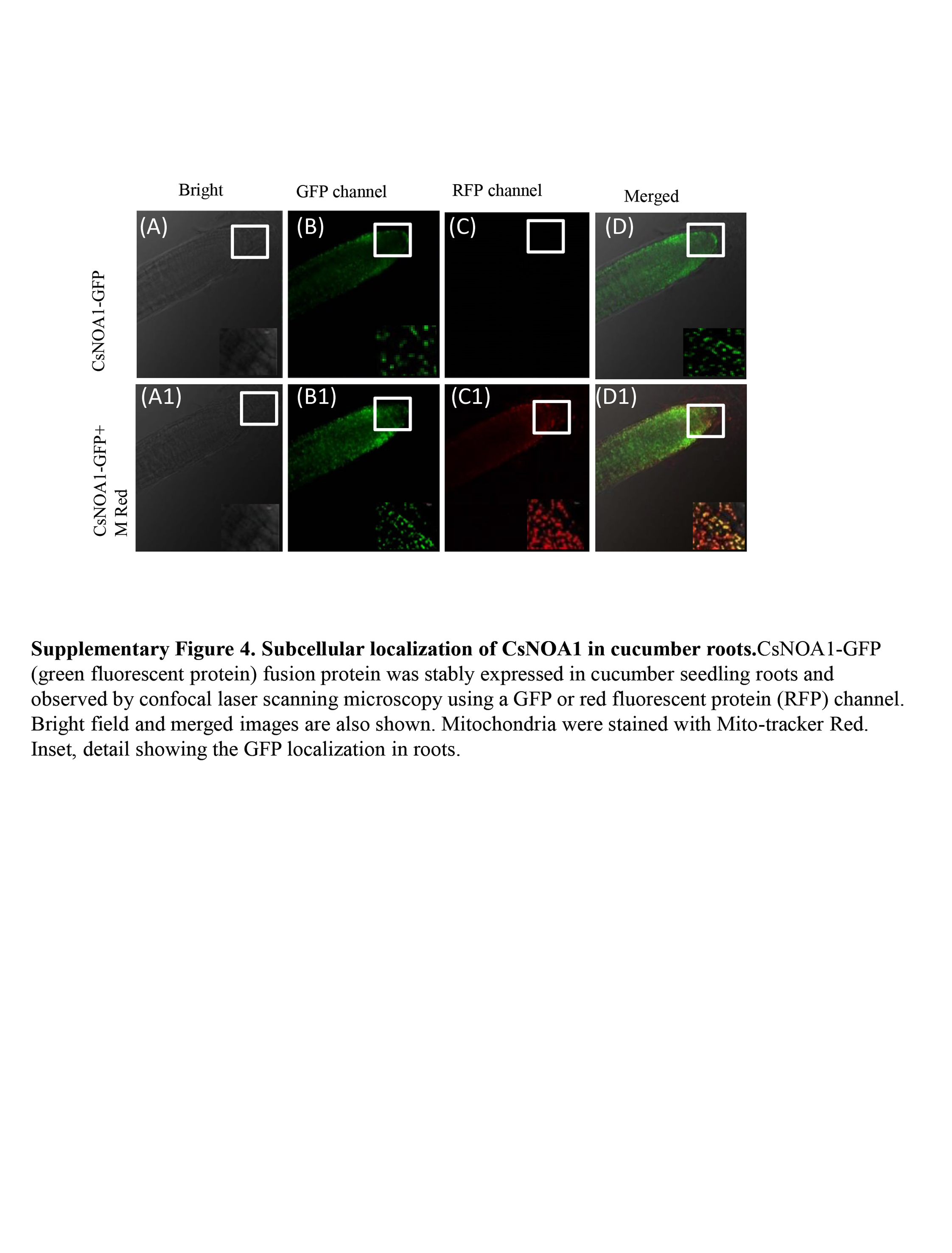

Supplement: Supplementary file 7 [file Image4.TIF]
